# Effects of Different Chili Pepper Varieties on the Quality and Microbial Diversity of Spontaneously Fermented Chili Paste

**DOI:** 10.3390/foods15111970

**Published:** 2026-06-02

**Authors:** Ke Li, Guangqin Zhang, Yurong Li, Yijie Dai, Jing Bai, Zongjun Li

**Affiliations:** 1 College of Food Science and Technology, Hunan Agricultural University, Changsha 410128, China; leeke2014@163.com (K.L.); zhang-guangqin@foxmail.com (G.Z.);; 2 Hunan Province Key Laboratory of Food Science and Biotechnology, Changsha 410128, China; 3 College of Biological and Environmental Engineering, Guiyang University, Guiyang 550005, China; dyj1003@163.com

**Keywords:** chopped chili peppers, chili pepper varieties, fermentation quality, spontaneously fermented chili paste

## Abstract

This study investigated how three chili pepper varieties (Huanggong, millet, and long slender) affect fermentation dynamics, flavor formation, and microbial succession in spontaneously fermented chopped chili. Physicochemical analyses, sensory evaluation, GC–MS, electronic tongue analysis, and high-throughput sequencing were employed to characterize quality attributes, volatile flavor profiles, and microbial structures during the process. The results demonstrated that chili pepper variety significantly influenced fermentation behavior (pH range: 4.07–4.26; total acidity: 5.68–7.69 g/kg) and quality. Distinct differences were observed in acidification patterns, substrate utilization, sensory characteristics, and optimal fermentation stages Volatile flavor analysis revealed that chopped chili peppers produced from different varieties exhibited differentiated aroma profiles while sharing a common flavor framework composed of several key aroma-active compounds. Microbial community analysis indicated that the chili pepper variety drove distinct microbial succession patterns during the process, and dominant microbial groups showed significant correlations with the formation of specific flavor compounds. Overall, these findings demonstrate that chili pepper variety regulates flavor and quality formation in chopped chili peppers by modulating microbial community structure and metabolic activity, providing a scientific basis for raw material selection and targeted flavor control in fermented chili products. A total of 63–68 volatile compounds were identified across varieties, with OAV > 1 for 11–24 compounds. Three biological replicates were analyzed per time point.

## 1. Introduction

Chili peppers (*Capsicum* spp.) are rich in bioactive compounds, including capsaicinoids, carotenoids, polyphenols, flavonoids, and various vitamins and minerals, which contribute to their antioxidant, antimicrobial, anticancer, and anti-obesity properties [1]. With the continuous expansion of chili-consuming populations, domestic chili consumption in China has maintained steady growth, revealing substantial market potential for deep-processed chili products [2]. Fermentation, as a key processing technology, not only extends the shelf life of chili peppers but also imparts unique sour and aromatic flavors while enhancing nutritional value through microbial metabolism [3]. Among the diverse array of traditional fermented chili products, chopped fermented chili (“duo la jiao”) is particularly favored by consumers due to its simple production process, distinctive spicy-sour flavor profile, and versatile culinary applications, most notably as a foundational seasoning in Hunan cuisine [4].

The quality and flavor of chopped fermented chili depend primarily on microbial community succession and metabolic activities during natural fermentation. This process occurs when raw peppers are fermented under saline conditions. Among the factors influencing this process, chili pepper variety serves as the fundamental determinant of fermentation substrate characteristics. Variations in nutritional composition (e.g., sugars, proteins, and volatile precursor compounds) and indigenous microbial populations across different varieties may significantly impact fermentation kinetics, final microbial community structure, and flavor quality [5,6]. Lactic acid bacteria (LAB), particularly *Weissella*, *Lactobacillus*, and *Pediococcus*, are the dominant functional microorganisms in chili fermentation. These bacteria drive acidification, produce antimicrobial compounds, and generate flavor precursors. Additionally, phytochemicals such as capsaicinoids and phenolic compounds exert selective pressure on microbial communities.

Although salinity is a critical environmental factor regulating fermentation, systematic scientific evidence guiding the selection of optimal salt concentrations tailored to specific chili varieties for achieving peak product quality remains limited. Furthermore, comprehensive investigations into how chili pepper varieties influence the production of fermented chopped chili are scarce, particularly regarding the underlying mechanisms by which different varieties shape unique microbial communities to drive the formation of characteristic flavor compounds. No study has systematically compared how different chili varieties, under identical salt conditions (10% *w*/*w*), drive divergent microbial successions and flavor formation. Specifically, the causal link between variety-specific substrate composition and the emergence of key microbial taxa (e.g., *Weissella*) remains unexplored. To address these knowledge gaps, this study employed three representative chili pepper varieties—Huanggong Jiao, Mi Jiao, and Xian Jiao—under a fixed salinity condition (10%, *w*/*w*) to systematically investigate (i) the differential effects of pepper variety on physicochemical properties, sensory quality, and dynamic changes in flavor compounds during fermentation; (ii) the succession patterns of microbial communities and their association with pepper variety; and (iii) the correlations between key microbial taxa and the formation of characteristic flavor compounds. The findings aim to elucidate the microbiological mechanisms by which pepper variety influences the fermentation quality and flavor development of chopped chili, thereby providing a theoretical basis for targeted production and process optimization of high-quality fermented chili products based on raw material characteristics.

## 2. Materials and Methods

### 2.1. Materials and Reagents

Table salt was sourced from a ocal supermarket; yellow bell peppers, long green peppers, and rice peppers were from Changsha Huangxing Haijixing International Agricultural Products Logistics Park. All peppers were harvested at commercial maturity (uniform red or yellow color), transported to the laboratory within 4 h post-harvest, and processed within 12 h. Storage was at 4 °C prior to processing. C7-C40 n-alkanes and 2-octanol standards were purchased from Sigma-Aldrich (St. Louis, MO, USA); acetonitrile, triethylamine, phenyl isothiocyanate, formic acid, n-hexane, and phosphoric acid (chromatography grade) were from McLean Biochemical Technology Co., Ltd. (Shanghai, China); oxalic acid, tartaric acid, malic acid, lactic acid, acetic acid, citric acid, succinic acid, fumaric acid, and other standard samples were purchased from Tanmo Quality Inspection Technology Co., Ltd. (Changzhou, China); and formaldehyde, sodium hydroxide, sodium chloride, hydrochloric acid, copper sulfate, potassium ferricyanide, zinc acetate, glacial acetic acid, and sodium metabisulfite (analytical grade) were from Sinopharm Chemical Reagent Co., Ltd. (Shanghai, China) Total DNA extraction and sequencing reagents for samples, along with the required instruments, were provided by Guangdong Meige Gene Technology Co., Ltd. (Shenzhen, China).

Details of the equipment used are as follows: DE-100 High-Speed Refrigerated Centrifuge, Hunan Hesi Instrument Equipment Co., Ltd. (Changsha, China); DZKW-S-8 Electric Heating Constant-Temperature Water Bath, Beijing Yongguangming Medical Instrument Co., Ltd. (Beijing, China); Synergy UV Ultrapure Water System, Wuhan Bailiezhen Biotechnology Co., Ltd. (Wuhan, China); ATY124 Electronic Analytical Balance, Shimadzu Management Co., Ltd. (Shanghai, China); PHS-3E pH Meter, Yidi Scientific Instrument Co., Ltd. (Taiwan, China); CM-2300D Color Difference Meter, Nanjing Kelipai Electronic Technology Co., Ltd. (Nanjing, China); WGL-125B Electric Heating Forced Air Drying Oven, Tianjin Test Instrument Co., Ltd. (Tianjin, China); Agilent 1260 High-Performance Liquid Chromatograph (Santa Clara, CA, USA), Agilent Technologies, Inc. (Santa Clara, CA, USA); TS-5000Z Electronic Tongue, Insent Co., Ltd. (Atsugi, Japan); 7000D Gas Chromatography–Mass Spectrometry System, Agilent Technologies, Inc. (Santa Clara, CA, USA); PC-420D Manual SPME Sampler, Supelco, Inc. (Bellefonte, PA, USA); 65 μm PDMS/DVB Extraction Fiber Head, Supelco, Inc. (Bellefonte, PA, USA); and HP-INNOWAX Capillary Chromatography Column, Agilent Technologies, Inc. (Santa Clara, CA, USA).

### 2.2. Methods

#### 2.2.1. Sample Preparation

We washed fresh yellow chili peppers (G10), M10 chili peppers, and X10 chili peppers. Fresh Huanggong, millet, and long slender peppers were washed, cut into chunks, mixed with 10% (*w*/*w*) salt, placed in clean fermentation bags, and fermented at 20 °C for 120 days. Initial microbial load was determined by plate counting before fermentation: aerobic bacteria ranged from 3.2 × 10^4^ to 5.8 × 10^4^ CFU/g, while yeasts and molds ranged from 1.5 × 10^3^ to 3.1 × 10^3^ CFU/g across varieties. Samples were retrieved at 0, 10, 20, 30, 60, 90, and 120 days for further analysis. Samples were stored at −20 °C for physicochemical parameter, organic acid, free amino acid, and volatile compound analyses. Huanggong Jiao is abbreviated as G; Mi Jiao as M; and Xian Jiao as X. Raw material and samples from days 0, 10, 20, 30, 60, 90, and 120 of fermentation were collected. Microbial diversity sequencing was performed by Guangdong Meige Gene Technology Co., Ltd. (Shenzhen, China) All submitted samples underwent three biological replicates to ensure accuracy.

#### 2.2.2. Determination of Basic Physical and Chemical Properties

pH Measurement: Accurately weigh 15 g of chopped chili sample, add 15 mL of ultrapure water, let stand for 10 min, and then measure using a pH meter. Repeat the measurement three times for each sample, and take the average as the final value. Total acid content shall be determined according to Method 1 of GB 12456-2021 [7]; total acidity was determined by NaOH titration and expressed as the lactic acid equivalent (g/kg). Amino acid nitrogen content shall be determined according to Method 1 of GB 5009.235-2016 [8]. Reducing sugars content shall be determined according to Method 1 of GB 5009.7-2016 [9].

The acidification during fermentation was primarily attributed to lactic acid fermentation, as confirmed by the detection of lactic acid and the proliferation of LAB (e.g., Weissella).

#### 2.2.3. Color Measurement

Color parameters of chopped chili samples were measured using a CM-2300d colorimeter, where L* represents lightness and darkness, a* represents redness, and b* represents yellowness and blueness.

#### 2.2.4. Sensory Evaluation

Sensory evaluation was conducted following the method of [10], with a maximum total score of 20 points. Five primary indicators—color, texture, aroma, crispness, and flavor—were selected, each weighted at 0.2. Ten professional sensory evaluators participated in the assessment, with scoring criteria detailed in Table 1.

#### 2.2.5. Electronic Tongue

Weigh 50 g of sample, add water to 259 mL, sonicate for 30 min, filter, and take 80 mL of the supernatant for electronic tongue data acquisition.

#### 2.2.6. Determination of Organic Acid Content

Refer to GB 5009.157-2016 [11], Determination of Organic Acids in Foods, with minor modifications. Mobile Phase: This consists of 0.1% phosphoric acid–water solution (Phase A) and 2.0% phosphoric acid–acetonitrile solution (Phase B). Elution Program: This consists of 2.5% B (0 min)−2.5% B (10 min)−100% B (15 min)−100% B (20 min)−2.5% B (25 min); temperature: 40 °C; and flow rate: 1.0 mL/min. Detect 7 organic acids at 210 nm wavelength, report results in mg/g, and qualitatively identify organic acids using standardized compounds.

#### 2.2.7. Determination of Free Amino Acid Content

Accurately weigh 10 g of chopped chili sample, add 30 mL water, boil in a water bath for 30 min, dilute to 50 mL, and filter. Accurately measure 500 μL of the hydrolyzed sample. Add 200 μL of triethylamine solution and 200 μL of phenyl isothiocyanate separately. Mix well and allow to stand at room temperature for 1 h for derivatization. After derivatization, add 1 mL of the lower layer solution and mix with 800 μL of water. Filter through a 0.22 μm organic-phase needle filter for injection.

#### 2.2.8. Determination of Volatile Aroma Compounds

Volatile compounds were extracted using headspace solid-phase microextraction (HS-SPME) following a modified method of [12]. Briefly, 2 g of sample was placed into a 20 mL headspace vial, and 4 mL of saturated NaCl solution was added. The vial was sealed and agitated at 80 °C for 10 min prior to SPME fiber exposure. The extraction needle was inserted, headspace adsorption was performed at 80 °C for 40 min, and then immediately the adsorbent-filled extraction head was inserted into the GC-MS inlet. This was desorbed at 250 °C for 5 min. Gas Chromatography Conditions: This comprised inlet temperature 230 °C; detector temperature 250 °C; carrier gas: high-purity helium (>99.99% purity); and flow rate 1.0 mL/min. Temperature Program: The initial column temperature of 40 °C held for 3 min, was ramped at 5 °C/min to 90 °C and then at 10 °C/min to 230 °C, and was finally held for 5 min. Mass Spectrometry Conditions: These included transfer port temperature 250 °C; inlet temperature 250 °C; ion source temperature 230 °C; electron energy 70 eV; and mass scan range m/z 35–400. Calculate the retention index (RI) of compounds based on the retention times of n-alkanes (C7-C40). Determine the concentration of volatile compounds using the internal standard method, with 2-octanol (0.0082 g/100 mL) as the internal standard. Compounds were identified by comparing results from the NIST 2017 database with mass spectra, combined with the retention time, retention index, and literature data.

Odor activity values (OAVs) are widely used to assess the contribution of certain flavor compounds. Compounds with OAV > 1 are typically considered aromatic active compounds, significantly contributing to the overall aroma profile of a sample. The OAV is calculated as the ratio of each VOC’s concentration (C) to its odor threshold (OT) in water. OT values were obtained from earlier reports using the formula: OAV = C/OT, where OAV is the compound’s odor activity value, C is the compound’s concentration, and OT is the compound’s odor threshold concentration in water.

#### 2.2.9. High-Throughput Sequencing Analysis

Microbial diversity sequencing was conducted by Guangdong Meige Gene Technology Co., Ltd. (Shenzhen, China), and all submitted samples underwent three biological replicates to ensure accuracy. The detailed procedure was as follows: Total DNA was extracted using the cetyltrimethylammonium bromide (CTAB) method and diluted to 1 ng/µL as template. The V3–V4 region of the bacterial 16S rRNA gene was amplified using barcoded specific primers 341F (5′-CCTACGGGNGGCWGCAG-3′) and 806R (5′-GGACTACHVGGGTWTCTAAT-3′). PCR products were mixed in equal proportions according to their concentrations, detected by 2% agarose gel electrophoresis, further purified using the AxyPrep DNA Gel Extraction Kit, and quantified with QuantiFluor-ST.

Libraries were constructed following the standard protocol of the ALFA-SEQ DNA Library Prep Kit. Library fragment sizes were assessed on the Qsep400 high-throughput nucleic acid and protein analysis system (Hangzhou Houze Biotechnology Co., Ltd., Hangzhou, China), and library concentrations were measured using a Qubit 4.0 fluorometer. The constructed amplicon libraries were subjected to PE250 sequencing on an Illumina or MGI platform.

After sequencing, raw reads were obtained and filtered to remove low-quality reads using FASTP v0.23.2 software. Paired-end reads were assembled into tags using Flash v1.2.11 software, followed by another round of filtering to obtain clean tags. Next, the UPARSE algorithm in USEARCH v11.0.667 software was used to cluster clean tags and remove chimeric tags detected during the clustering process, yielding effective tags. OTU (Operational Taxonomic Unit) abundance statistics were ultimately calculated based on the effective tags.

#### 2.2.10. Data Processing

Basic data processing was performed using Excel, with results presented as the “mean ± standard deviation.” SPSS statistical software (IBM SPSS Statistics 27.0) was employed for significance analysis and Spearman correlation analysis between the top 10 microbial species by relative abundance during fermentation stages of different chili varieties and key volatile flavor compounds in each sample group. Origin 2021 was employed for graphing. Statistical analysis was conducted using software such as Meige Gene’s cloud analysis and cloud tool platforms (latest version). Graphing was performed using cloud tools from the Maiwei Cloud Platform.

## 3. Results and Discussion

### 3.1. Effects of Different Pepper Varieties on Physicochemical Indicators During Fermentation

**Figure 1:**
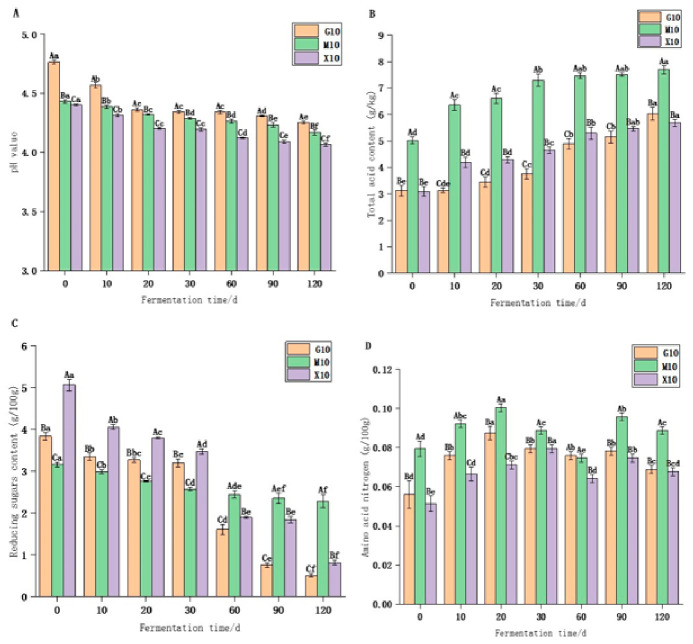
Changes in pH (**A**), total acidity (**B**), reducing sugars content (**C**), and amino acid nitrogen content (**D**) of chopped peppers of different varieties during the fermentation process. Data are presented as the mean ± standard deviation (n = 3). Different uppercase letters (A, B, C) denote significant differences (*p* < 0.05) among the three groups at the same fermentation time point. Different lowercase letters (a, b, c, d, e, f) denote significant differences (*p* < 0.05) across fermentation times within the same group.

Figure 1 shows dynamic changes in pH, total acidity, reducing sugars, and amino nitrogen across varieties. These changes reflect variations in microbial metabolic activity and substrate conversion. The three varieties exhibited distinct fermentation kinetic profiles: the long chili pepper belonged to the “rapid acidification type,” the rice pepper to the “high substrate reserve type,” and the yellow Gong chili pepper to the “late acid accumulation-driven type.”

Figure 1A illustrates that the pH of all varieties decreased throughout fermentation. At the end of fermentation, the long chili pepper had the lowest pH at 4.07, followed by the rice pepper at 4.17 and the yellow Gong chili pepper at 4.26. The pH decline rate of Huanggong peppers was significantly higher than the other two groups (*p* < 0.05). This rapid acidification is attributed to the proliferation of *Weissella*, which reached 89.94% relative abundance by day 120. Changes in total acid content (Figure 1B) showed that yellow Gong peppers increased from 3.12 g/kg to 6.03 g/kg, with an absolute increment significantly higher than both rice peppers (5.02–7.69 g/kg) and long peppers (3.08–5.68 g/kg) (*p* < 0.05), indicating more pronounced organic acid accumulation during the mid-to-late fermentation stages. This is attributed to acid-producing metabolism by dominant later-stage microbial communities, such as *Weissella* species.

The dynamic changes in reducing sugars content serve as an important indicator for evaluating microbial carbohydrate utilization during fermentation [13]. Changes in reducing sugars content reflect microbial utilization of carbohydrates (Figure 1C). Chili peppers exhibited the highest initial reducing sugar levels and fastest consumption rate yet showed the lowest total acid accumulation. Huanggong peppers displayed moderate initial sugar content but the most pronounced acid accumulation, suggesting that beyond substrate levels, microbial community structure significantly regulates carbon source utilization efficiency and acid production pathways.

Changes in amino acid nitrogen are shown in Figure 1D. Rice peppers maintained the highest levels throughout fermentation and exhibited relatively high residual total acid and reducing sugar, indicating that their raw material is rich in both protein and carbohydrates. This provided microorganisms with a sustained and balanced nutrient substrate, thereby influencing microbial community succession and metabolic pathways.

### 3.2. Effect of Different Chili Pepper Varieties on Color During Fermentation of Minced Chili Peppers

Table 2 presents the L* values of minced chili peppers from all three varieties decreased with extended fermentation time. The Huanggong Jiao group decreased from 51.03 to 44.99; the Mi Jiao group decreased from 45.66 to 42.4; and the long pepper group decreased from 44.63 to 39.78. The decrease in the Huanggong Jiao group was significantly greater than that of the other two groups (*p* < 0.05), and the decrease in the Mi Jiao group was the smallest, indicating a lower degree of browning. During the process, color intensity darkened, closely related to the Maillard reaction between reducing sugars and amino compounds in peppers, as well as polyphenol oxidase-catalyzed browning reactions [14].

The a* value of the Huanggong Jiao group decreased from 10.52 at the start of fermentation to 8.67; the Mi Jiao group decreased from 29.72 to 21.74; and the long pepper group decreased from 27.42 to 17.69. The decrease in a* value for the long pepper group was significantly greater than the other two groups (*p* < 0.05). At the end of fermentation, the rice pepper group had the highest a* value, and the yellow Gong pepper group had the lowest. Regarding the b* value, the yellow Gong pepper group decreased from 30.76 to 20.98; the rice pepper group decreased from 19.76 to 15.81; and the long pepper group decreased from 16.65 to 13.2. The decrease in b* value for the yellow gong pepper group was significantly greater than that of the other two groups (*p* < 0.05). These color changes may be related to the microbial degradation of pigments such as carotenoids in pepper varieties, leading to a lightening of red and a weakening of yellow hues [15].

Color changes showed good consistency with the color item in sensory evaluations. For example, the Huanggong chili exhibited the largest decrease in L value, corresponding to its lowest sensory color score; the Mi chili had the highest a value and a smaller decrease in L value, confirming its description as having the best color stability in sensory evaluations. These variations in browning intensity primarily stem from two factors: first, differences in the composition and content of intrinsic pigments (e.g., carotenoids and anthocyanins) within each variety; and second, variations in the degradation rates of pigment compounds due to microbial metabolism and enzymatic reactions during the process. The Maillard reaction and oxidation of phenolic compounds likely represent common pathways contributing to the widespread decrease in L-value across all varieties.

### 3.3. Effect of Different Chili Pepper Varieties on Sensory Scores During Fermented Minced Chili Processing

**Figure 2:**
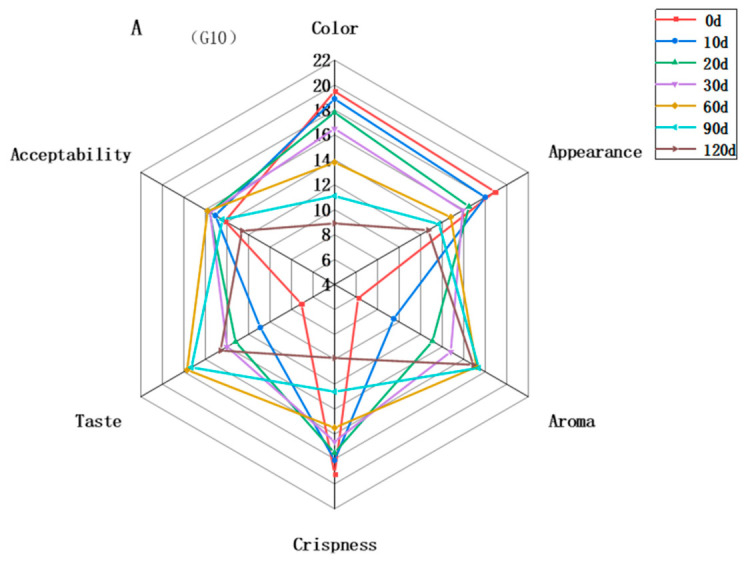

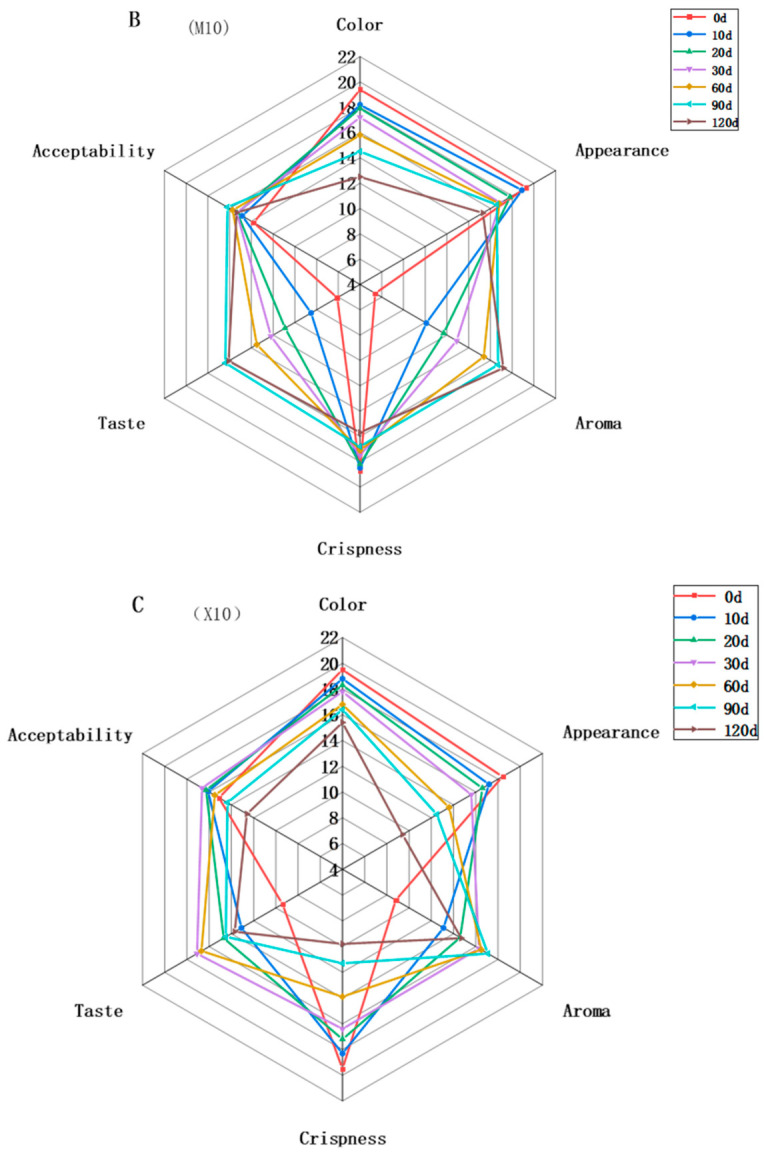
Radar chart of sensory evaluation scores for different varieties of chopped chili peppers during the process: (**A**) Huanggong pepper; (**B**) millet pepper; (**C**) long slender pepper.

The changes in sensory scores for the three varieties of chopped chili during the process are shown in Figure 2. Overall, as the fermentation period lengthened, the scores for color, crispness, and texture declined across all varieties. In the early stages of fermentation, the chopped chilis exhibited characteristics of raw spiciness, with no acidity or aroma; subsequently, they gradually developed an optimal flavor profile characterized by a rich aroma, a mellow taste, appropriate acidity, and a mild spiciness. However, excessive fermentation can easily lead to the proliferation of off-flavor-causing bacteria, resulting in excessive acidity and flavor deterioration, accompanied by quality degradation such as a dull color, reduced crispness, and increased separation of skin and flesh [16].

Based on the sensory scoring radar chart (Figure 2) and data at the end of fermentation, the three varieties exhibited significant differences across all indicators. In terms of color, the Huanggong Jiao group received the lowest score, whilst the Linear Jiao group received the highest, indicating that the Linear Jiao group demonstrated better color stability during the process, which may be related to the greater resistance of its pigment components. In terms of crispness and morphology, the Mi Jiao group received the highest score, suggesting that it maintained the integrity of its tissue structure well during the process; conversely, the Long Jiao group received the lowest score for morphology, which may be due to the degradation of pectin and a decrease in turgor pressure under the action of microorganisms, leading to tissue softening [17].

Regarding aroma, both the Yellow Gongjiao and Threaded Chili groups exhibited an initial rise followed by a decline, peaking on the 90th day of fermentation at 17.4 and 17.1 points, respectively; the Mi Jiao group, however, achieved its highest aroma score (17.2 points) towards the end of fermentation, demonstrating good aroma persistence. During the early fermentation stage (0–30 days), the aroma and flavor scores of the thread pepper group were significantly higher than those of the other two groups; this is related to the abundant volatile aromatic compounds and flavor compounds produced in the early stages, although some of these compounds volatilized or were metabolized by microorganisms in the later stages [18].

Overall acceptability comprehensively reflects consumers’ preference for chopped chili. Combined with the fact that the overall acceptability of the G10, M10, and X10 groups was 100 in the early fermentation stage (as shown in Figure 2), this indicates that all three varieties had a high baseline of consumer acceptability at the outset. During the 0–30-day fermentation period, the overall acceptability of the long chili group was higher than that of the other two groups, with the highest score recorded at day 30 (16.64 points); the Huanggong Jiao group achieved the highest score at 60 days of fermentation (15.8 points), whilst during the 90–120-day fermentation period, the Mi Jiao group’s overall acceptability was higher than that of the other two groups, with the highest score recorded at 90 days of fermentation (16.2 points). This indicates that different varieties exhibit distinct periods of advantage at different fermentation stages: thread chilis are suitable for short-term fermentation, whilst rice chilis perform better in long-term fermentation.

### 3.4. Analysis of the Impact of Different Chili Pepper Varieties on Taste Characteristics During Fermented Chili Paste Production Using an Electronic Tongue

**Figure 3:**
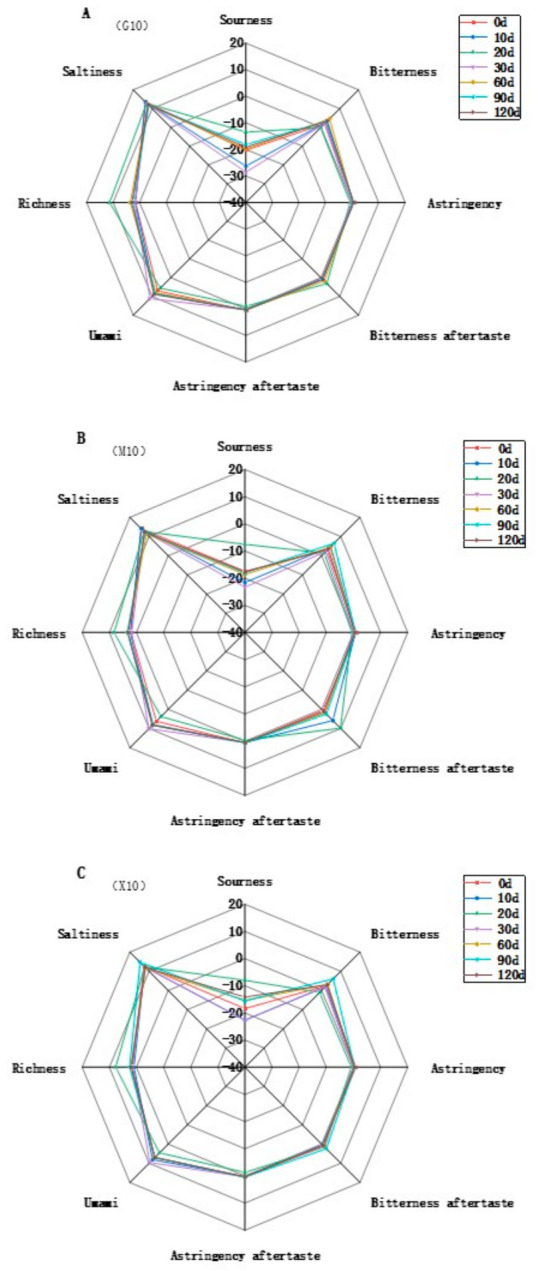
Radar chart of electronic tongue sensor responses for different varieties of chopped chili peppers during the process: (**A**) Huanggong pepper; (**B**) millet pepper; (**C**) long slender pepper.

An electronic tongue enables digital and objective evaluation of fundamental taste attributes in food, including sourness, bitterness, astringency, saltiness, and umami [19]. If the response value for a particular taste (e.g., sourness or umami) falls below the taste threshold, it indicates that this taste characteristic is either absent or extremely weak in the sample [20]. The sourness response values for all three chopped chili pepper varieties were below the taste threshold, suggesting that sourness was not perceived during the process. As shown in Figure 3, the saltiness response values were the highest among the three varieties of chopped chili peppers, followed by umami response values, indicating that their taste characteristics were predominantly dominated by saltiness and umami. The umami response values for the Huanggong Jiao, Mi Jiao, and Xian Jiao groups exhibited dynamic trends, all peaking on the 30th day of fermentation. The highest umami response values for Huanggong Jiao, Mi Jiao, and Xian Jiao were 11.14, 10.06, and 9.61, respectively. The umami response values for Huanggong Jiao, Mi Jiao, and Xian Jiao reached their lowest point on the 20th day of fermentation. Among these, the three indicators for Huanggong Jiao and Xian Jiao were below the taste threshold; for the Mi Jiao group, both the astringency and astringency aftertaste response values were below the taste threshold, with the bitterness response value being the lowest at 1.36. Regarding richness response values, the Huanggong chili, rice chili, and long chili groups all reached their highest values on day 20 at 11.33, 8.24, and 7.64, respectively. Throughout the fermentation process, the aftertaste bitterness response value of the Mi Jiao group consistently exceeded that of the other two groups, peaking at 9.81 on day 20. The bitterness response values for the Huanggong Jiao and Xian Jiao groups reached their highest levels on days 20 and 90, respectively, at 3.1 and 2.44.

### 3.5. Effects of Different Chili Pepper Varieties on Organic Acid Content During the Processing of Minced Chili Peppers

**Figure 4:**
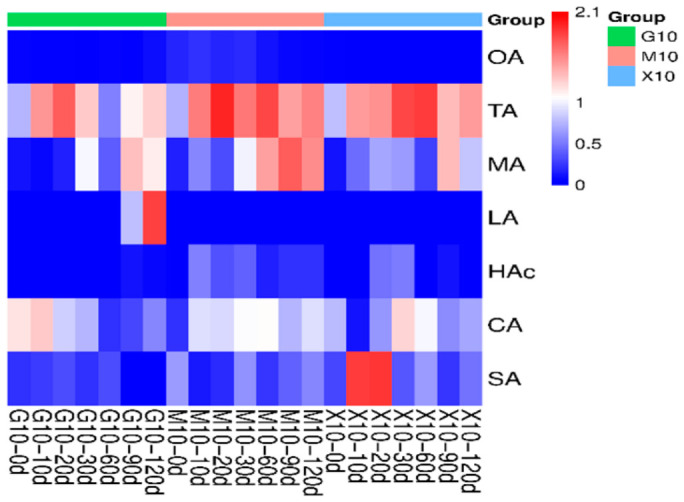
Heatmap of organic acid contents in different varieties of chopped chili peppers during the process.

The organic acid content of minced chili peppers of different varieties exhibits complex changes during the process. The variation in organic acid content for different varieties is shown in Tables S1–S3, with the heatmap of organic acid content presented in Figure 4.

The organic acid content of chopped chili peppers of different varieties exhibited distinct changes during the process, with significant differences observed between varieties. Oxalic acid remained at low levels across all three samples, while tartaric acid, malic acid, and citric acid constituted the primary organic acid components. During the 90–120 d fermentation period, the Mi Jiao group exhibited the highest tartaric acid content, followed by the Huanggong Jiao and Xian Jiao groups. The tartaric acid content in the Mi Jiao group showed the most significant change before and after fermentation, increasing from 0.725 mg/g to 1.588 mg/g. Malic acid, characterized by a mild acidity, is one of the key organic acids in microbial metabolic processes [21]. From days 60 to 120 of fermentation, the Mi Jiao group consistently exhibited higher malic acid levels than the other two groups. The Huanggong Jiao group showed significantly higher citric acid levels than the other two groups during the early fermentation stage (days 0–10). Lactic acid possesses a mild sour taste and is a crucial organic acid in fermented foods [22]. Throughout the fermentation period, neither lactic acid nor acetic acid was detected in samples from the rice pepper or long pepper groups. Lactic acid and acetic acid were first detected in samples from the yellow Gong pepper group at day 60 of fermentation, with lactic acid levels exceeding those of the other two groups by day 120. At the end of fermentation, the total organic acid content was highest in the rice pepper group, followed by the yellow gong pepper group and the long pepper group. Furthermore, the content of each organic acid in the rice pepper group was higher than in the other two groups (except for lactic acid).

### 3.6. Effects of Different Chili Pepper Varieties on Free Amino Acid Content During Fermented Chili Paste Production

**Figure 5:**
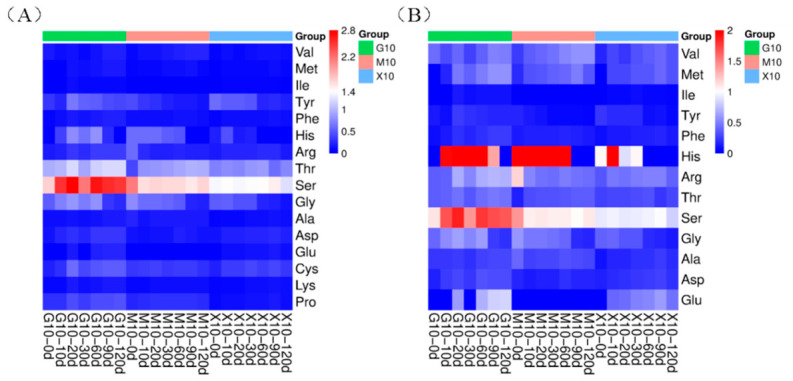
Changes in free amino acid contents during the fermentation process of different chopped chili pepper varieties: (**A**) content heatmap; (**B**) taste activity value (TAV) heatmap.

Free amino acids serve both nutritional and flavor-modulating functions. Among them, aspartic acid and glutamic acid are umami amino acids; seven amino acids including histidine, arginine, and valine are classified as bitter amino acids, while four amino acids such as serine and threonine belong to sweet amino acids [23]. Their content changes are closely related to microbial metabolism and the hydrolytic action of endogenous proteases [24]. The changes in the content of 16 free amino acids and their taste activity values (TAVs) are shown in Heatmap 5 (specific contents are listed in Tables S4–S6). According to the definition of taste activity value, when TAV > 1, the corresponding free amino acid is considered an active substance significantly contributing to food flavor [25]. As shown in Figure 5A, serine is the primary free amino acid in all three chopped chili pepper samples. Among them, the Huanggong Jiao group exhibited the highest serine content throughout the fermentation process, while the Xian Jiao group had the lowest. As shown in Tables S4–S6, neither isoleucine nor glutamic acid was detected in the rice pepper group throughout fermentation. Isoleucine and glutamic acid appeared in the long pepper group starting on day 10 of fermentation, while they were first detected in the yellow Gong pepper group on day 20. From the amino acid flavor profile analysis, sweet amino acids were the most abundant throughout fermentation in all three chopped chili samples, followed by bitter amino acids, while umami amino acids were the least abundant. Between days 60 and 120 of fermentation, the Huanggong chili group exhibited the highest levels of sweet and umami amino acids, while the rice chili group had the lowest umami amino acid content. In summary, the results indicate that Huanggong Jiao significantly enhances the umami and sweetness intensity of chopped chili peppers after 60 days of fermentation, closely related to the dynamic changes in its free amino acid composition and content. As shown in Figure 5B, serine contributed to the flavor profile of all three chopped chili varieties throughout fermentation, with the strongest effect observed in the Huanggong Jiao group, followed by the Mi Jiao group and finally the Xian Jiao group. From days 10 to 60 of fermentation, histidine contributed more to the flavor of the Huanggong Jiao variety than serine (except on day 90). At day 0 of fermentation, histidine, arginine, and threonine all contributed to the flavor profile of the rice pepper group, with histidine playing a more significant role than the other two amino acids. However, histidine’s contribution to the flavor of both the rice pepper and long pepper groups was only maintained during the fermentation period from day 0 to day 60.

### 3.7. Changes and Analysis of Volatile Compounds During the Fermentation of Different Varieties of Chopped Chili

**Figure 6:**
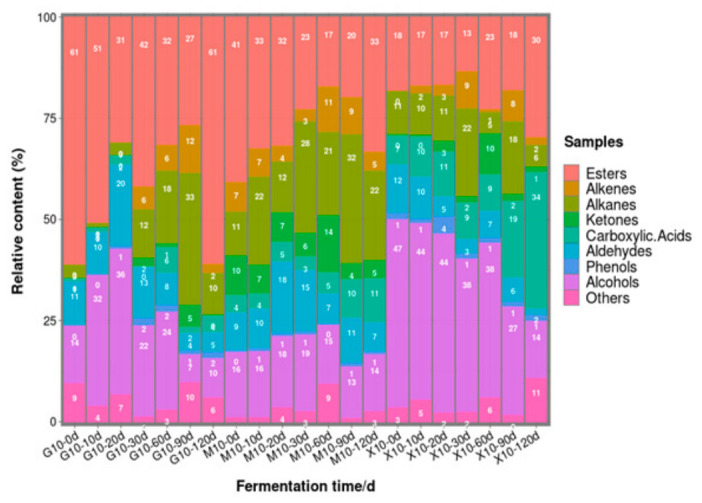
Changes in total volatile flavor compounds during the processing of different chopped chili pepper varieties.

GC-MS technology was employed to determine volatile compounds during the fermentation of chopped chili peppers of different varieties. The Huanggong Jiao group, Mi Jiao group, and Xian Jiao group yielded 65, 63, and 68 flavor compounds, respectively, as shown in Tables S7–S9. Figure 6 illustrates the differences in volatile flavor compound categories. As shown in Figure 6, significant amounts of ester compounds, alcohol compounds, aldehyde compounds, acid compounds, ketone compounds, alkane compounds, alkene compounds, and heterocyclic compounds were detected during the fermentation of all chili pepper groups. The Huanggong Jiao group yielded 20 esters, 17 alcohols, 6 alkenes, 6 alkanes, 4 aldehydes, 4 other compounds, 3 ketones, 3 acids, and 2 phenols; esters constituted its primary flavor compounds. The rice pepper group contained 17 esters, 12 aldehydes, 10 other compounds, 9 alkenes, 8 alcohols, alkanes (8 types), acids (8 types), ketones (5 types), and phenols (3 types). Alkane compounds were relatively high during the 30–90-day fermentation period, while ester compounds reached higher levels at the end of fermentation. In the long pepper group, 21 esters, 16 alcohols, 12 aldehydes, 10 acids, 8 other compounds, 7 alkanes, 6 alkenes, 5 phenols, and 4 ketones were detected. Alcohol compounds dominated significantly during the 0–90-day fermentation period, while acid compounds reached peak levels at the end of fermentation.

**Figure 7:**
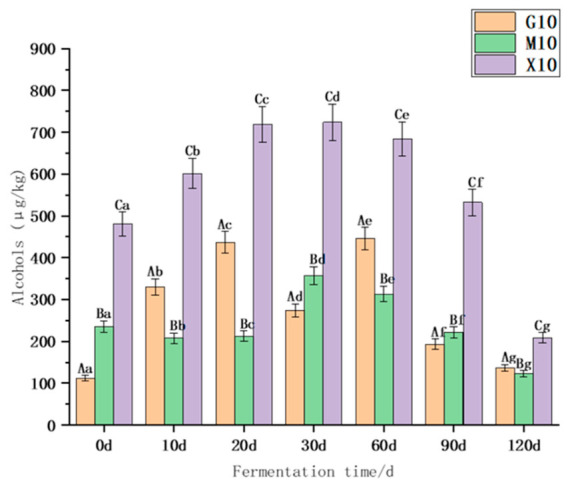
Changes in alcohol compound contents during the processing of different chopped chili pepper varieties. Values are expressed as the mean ± standard deviation (n = 3). Different uppercase letters (A, B, C) indicate significant differences (*p* < 0.05) among the three groups at the same fermentation time point. Different lowercase letters (a, b, c, d, e, f, g) indicate significant differences (*p* < 0.05) across fermentation times within the same group.

Alcohol compounds, as precursors to aromatic esters, not only drive the formation of esters in chopped chili peppers but also impart floral and fruity aromas, making them one of the key volatile flavor components in chopped chili peppers [26,27]. Alcohol compounds primarily originate from microbial-mediated sugar metabolism pathways [28], redox pathways involving unsaturated aldehydes or ketones [29], and amino acid metabolism pathways [30]. Figure 7 shows the changes in alcohol content during the fermentation of chopped chili peppers of different varieties. Between 60 and 120 days of fermentation, the ester content in all three varieties gradually decreased with prolonged fermentation time. This decline may result from the decreasing pH in the fermentation system, where the acidic environment promotes esterification reactions between alcohols and organic acids, leading to ester formation and consequently reduced alcohol content [31].

The total alcohol content of the long chili was significantly higher than the other two groups, exhibiting a dynamic pattern of initial increase followed by decrease in the process. The predominant alcohols varied among varieties: Phenethyl alcohol (rose-like sweet aroma) and 4-methyl-1-pentanol (woodsy, green scent) were common components, with the long chili containing the highest phenethyl alcohol and the rice chili containing the highest 4-methyl-1-pentanol. Additionally, the higher levels of pinene (lilac and lily-of-the-valley aroma) and n-hexanol (lily, rose, and fatty aroma) in long peppers contributed to a richer aromatic profile. Rice peppers, on the other hand, contained more nerol, imparting rose and apple aromas.

Aldehydes impart the distinctive flavor profile of chopped chili peppers. For instance, unsaturated aldehydes often carry fresh grassy, fruity, or nutty aromas [32], while saturated aldehydes exhibit creamy or floral scents [33]. Aldehyde formation is associated with the oxidation of unsaturated fatty acids [34], amino acid metabolism [35], and the Maillard reaction [36].

**Figure 8:**
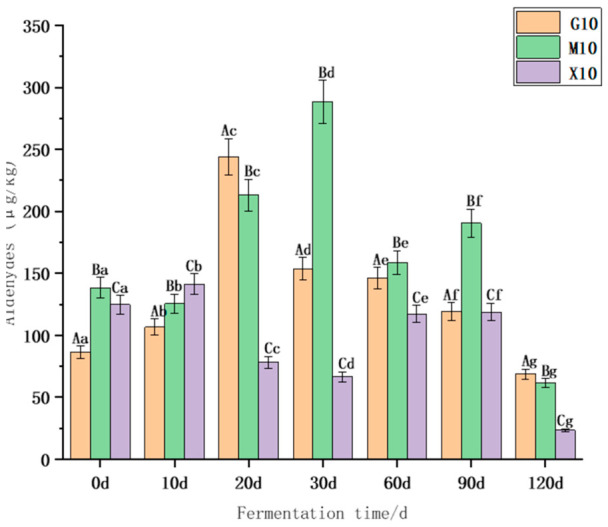
Changes in aldehyde compound contents during the processing of different chopped chili pepper varieties. Values are expressed as the mean ± standard deviation (n = 3). Different uppercase letters (A, B, C) indicate significant differences (*p* < 0.05) among the three groups at the same fermentation time point. Different lowercase letters (a, b, c, d, e, f, g) indicate significant differences (*p* < 0.05) across fermentation times within the same group.

Figure 8 illustrates the changes in aldehyde content during the fermentation of chopped chili peppers of different varieties. All three varieties exhibited dynamic shifts in aldehyde levels throughout fermentation, reflecting the tendency of aldehydes to be reduced into corresponding alcohols or acids during this process [37]. At the end of fermentation, aldehyde levels in all three varieties were significantly lower than in earlier stages. The Huanggong Jiao group exhibited higher levels of trans-2-nonanal and phenylacetaldehyde, contributing to its rich oily aroma and hyacinth-like floral notes. The Mi Jiao group exhibited significant levels of (2E,4E)-dec-2,4-dienal and trans-2-octenoic acid, whose combined cucumber-like freshness and oily aroma constituted the group’s distinctive flavor profile. The Xian Jiao group showed the lowest aldehyde content at the end of fermentation, at only 23.27 μg/kg, significantly lower than the other two groups.

Esters not only impart the distinctive floral and fruity aromas to chopped chili peppers but also effectively mitigate the pungent odors produced by excessive concentrations of medium-chain fatty acids, rendering the overall flavor profile more mellow and harmonious [38]. Most esters are formed through esterase-catalyzed reactions between alcohols and acids derived from glucose and amino acids during microbial metabolism [39] or through biosynthesis using alcohol acetyltransferase with alcohols and acetyl-CoA as substrates [40].

**Figure 9:**
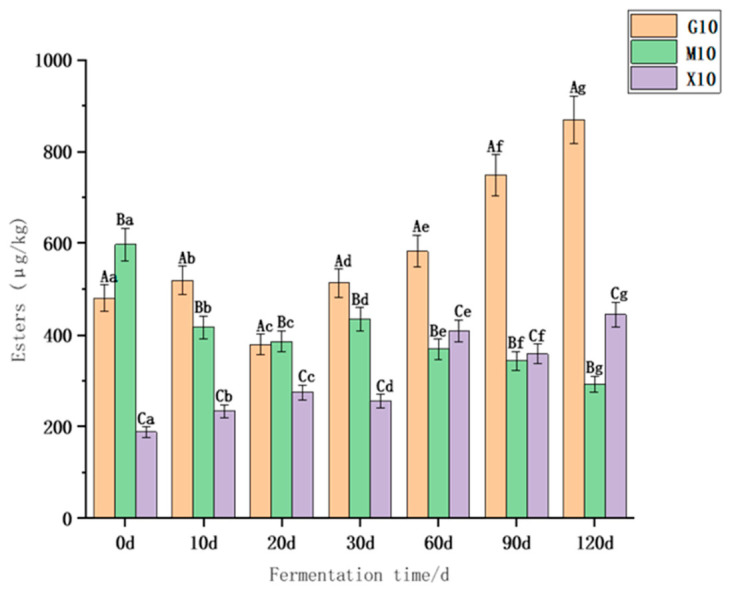
Changes in ester content during the processing of different varieties of chopped chili peppers. Values are expressed as the mean ± standard deviation (n = 3). Different uppercase letters (A, B, C) indicate significant differences (*p* < 0.05) among the three groups at the same fermentation time point. Different lowercase letters (a, b, c, d, e, f, g) indicate significant differences (*p* < 0.05) across fermentation times within the same group.

Changes in ester content during the fermentation of different chopped chili pepper varieties are shown in Figure 9. The trends in ester content in the process differed significantly among the three varieties. Ester levels in the Huanggong Jiao group samples increased continuously in the process (30–120 days) and remained higher than in the other two groups. Methyl salicylate was the primary ester present throughout fermentation in all three varieties. Possessing a fresh, mild fragrance reminiscent of wintergreen leaves, its content significantly decreased after fermentation. The Mijiao group exhibited higher methyl salicylate levels than the other two groups, ranging from 91.57 to 485.55 μg/kg. Ethyl hexadecanoate was a major ester common to all three chopped chili pepper varieties during the 60~120-day fermentation period, with the highest content in the Huanggong Jiao group. This compound possesses subtle wax, fruity, and creamy aromas, contributing to a richer flavor profile in the chopped chili peppers. Additionally, post-fermentation levels significantly increased for ethyl linoleate, ethyl linolenate, and ethyl caproate in the Huanggong Jiao group; ethyl acetate in the Long Pepper group; and ethyl nonanoate in the Rice Pepper group. Except for ethyl tetradecanoate, which imparts a wine-like aroma to chopped chili peppers, these esters collectively contribute to the floral and fruity notes in fermented chili peppers.

### 3.8. Partial Least Squares (PLS) Analysis

**Figure 10:**
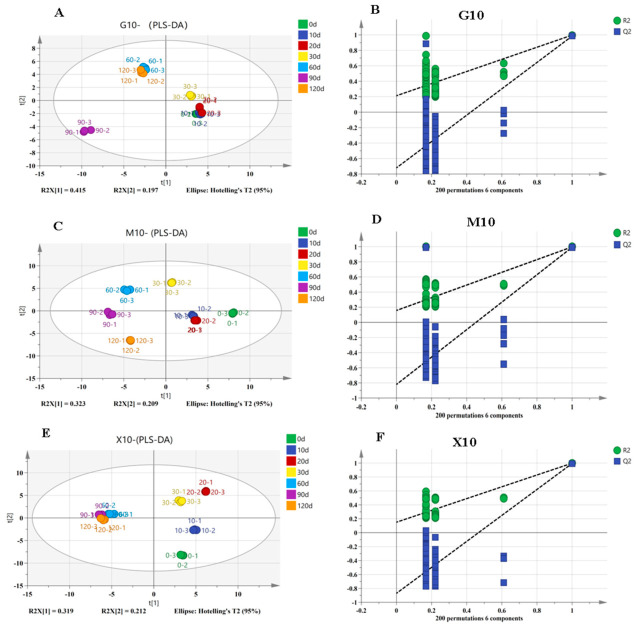
PLS-DA Score Plots (**A**,**C**,**E**) and Permutation Plots (**B**,**D**,**F**) of chopped chili peppers of different varieties.

Partial Least Squares Discriminant Analysis (PLS-DA) is a statistical method that reduces dimensionality to visualize complex data for discrimination [41]. To evaluate differences in volatile flavor compounds between individual or groups of chopped chili varieties in the process, the results are shown in Figure 10. In the OPLS-DA model, both R^2^X and Q^2^ exceeded 0.9, indicating model suitability. Cross-validation analysis with 200 permutations confirmed no overfitting, demonstrating robust data quality.

In the Huanggong Jiao group, samples from days 0 to 30 showed significant clustering, primarily in the first and fourth quadrants. Samples from days 60 and 120 were located in the second quadrant. The day 90 sample was isolated in the third quadrant, distant from samples of other days, indicating similar volatile flavor compound compositions in Huanggong Jiao samples during the early fermentation stage (days 0~30). In the Mi Jiao group, samples from days 10 and 20 showed pronounced clustering, while samples from other fermentation days exhibited relatively dispersed distributions. This indicates that the flavor compound composition of Mi Jiao remained relatively stable during the early fermentation stage, with significant changes occurring in the volatile flavor compound composition as fermentation time progressed. In the long chili pepper group, samples from 60, 90, and 120 days showed some overlap, while samples from other fermentation days were relatively dispersed. This indicates that after 60 days of fermentation, the overall changes in volatile flavor compounds in the long chili pepper group were relatively minor.

To identify key odor components influencing the flavor profiles of different chili pepper varieties, an evaluation was conducted by calculating the odor activity value (OAV, defined as the ratio of concentration to odor threshold) for each volatile compound. Typically, compounds with OAV > 1 are considered to make a significant contribution to the overall flavor [42]. The results are presented in Table 3, Table 4 and Table 5.

Eleven, seventeen, and twenty-four compounds with OAV > 1 were identified in Huanggong Jiao, Mi Jiao, and Xian Jiao, respectively. Ethyl caproate was identified as a key compound specific to Huanggong Jiao, exhibiting pineapple, apple, and floral characteristics, with the highest OAV (3.70) observed at 90 days of fermentation [43]. The key compounds specific to Mi peppers are cyclopentadecanolide and violone, both exhibiting floral notes [44]. Key compounds unique to long peppers include trans-2,4-decadienal, ethyl hexanoate, ethyl decanoate, and n-pentanoic acid. Among these, trans-2,4-decadienal and ethyl hexanoate primarily contribute during the mid-to-late fermentation stages (60–120 days).

Hexanol (apple and banana aroma) is a key contributor throughout all stages except the final stage, with OAVs ranging from 19.93 to 31.50. Six key flavor compounds are common to all three varieties: methyl salicylate, caryophyllene, β-ionone, β-farnesone, trans-2-nonanal, and linalool. Their contribution levels vary across varieties. For instance, at fermentation completion, β-ionone exhibits an exceptionally high OAV of 3670.51 in Mi Jiao, delivering pronounced violet floral notes accompanied by woody and fruity aromas [45]. Methyl salicylate and linalool achieve their highest OAVs in Mi Jiao and Xian Jiao, respectively.

In summary, the three varieties exhibit distinct flavor profiles (Table 3, Table 4 and Table 5): the Huanggong Jiao variety is characterized by spiciness (cinnamaldehyde), floral/fruity notes (eugenol and linalool), and prominent grassy/fatty aromas (trans-2-nonenal and (2E,4E)-dec-2,4-dienal). floral notes (ethyl caprate and linalool), and pronounced grassy/fatty aromas (trans-2-nonanal, (2E,4E)-dec-2,4-dienal), creating a rich and complex flavor profile; the Mijiao chili is dominated by intense floral/woody notes (β-ionone), complemented by green notes (trans-2-octenaldehyde) and fruity notes (cyclopentadecanol), presenting a distinct profile; and the long pepper chili exhibits fresh floral/fruity notes (linalool and n-hexanol) and grassy notes (trans-2-octenaldehyde), with a relatively mild flavor. These characteristic flavor profiles arise from differences in precursor compounds among varieties and the specific microbial metabolic activities they drive.

During the fermentation process of the Huanggong pepper group, the ROAV changes exhibited distinct characteristics (Table 6). At the early fermentation stage (0 days), (2E,4E)-deca-2,4-dienal had an ROAV of 100.00, and trans-2-nonenal had an ROAV of 52.56, indicating that C10 and C9 alkenals dominated the aroma, presenting fatty and green notes. From 10 d to 60 days, (2E,4E)-deca-2,4-dienal remained at 100.00, while trans-2-nonenal generally decreased, and its aroma contribution weakened. By 90 days, β-damascenone’s ROAV increased to 100.00 for the first time, replacing the former (which decreased to 13.52) as the new dominant component, and the aroma shifted to floral and fruity notes. At 120 days, β-damascenone was not detected, (2E,4E)-deca-2,4-dienal decreased to 9.43, and β-ionone was only 0.09, indicating a significant decrease in overall aroma intensity. The ROAVs of linalool and methyl salicylate remained consistently low (maximum 2.07 and 1.52, respectively), contributing little. Overall, the Huanggong pepper group completed the replacement of dominant aroma components in the middle of fermentation, and the aroma gradually transitioned from fatty and green notes to floral notes.

Compared with the Huanggong pepper group, the aroma composition of the millet pepper group was highly stable (Table 7). From 0 d to 120 days, the ROAV of β-damascenone was always 100.00, making it the absolutely dominant component with a consistently strong contribution. β-Damascenone has typical floral, fruity, and sweet characteristics; therefore, the aroma of the millet pepper group was predominantly floral throughout the process. In the millet pepper group, (2E,4E)-deca-2,4-dienal was detected from 10 days onwards and gradually increased, reaching a maximum of 58.14 at 60 days, and then decreased to 36–42, providing some fatty and green notes. The ROAVs of trans-2-nonenal, trans-2-octenal, linalool, and methyl salicylate did not exceed 4.00 and were mostly below 1.00, contributing little. 2-Methoxy-3-isobutylpyrazine appeared at 20 days and 120 days with ROAVs of 0.51 and 0.62, respectively, bringing a slight bell pepper note. Overall, the aroma of the millet pepper group was highly consistent, with β-damascenone always dominating and other components only playing auxiliary and modifying roles.

The aroma changes in the long slender pepper group were the most complex, showing distinct early, middle, and late stages (Table 8). At the early fermentation stage (0 days), linalool had an ROAV of 100.00, 1-hexanol was as high as 120.07 (possibly a data labeling issue), and nonanal and hexanal were 48.53 and 23.18, respectively, presenting fresh, green, and fruity characteristics. At 10 days, linalool remained at 100.00, while 2-methoxy-3-isobutylpyrazine rapidly increased to 76.43, and trans-2-nonenal also increased to 69.53, indicating a significant enhancement of bell pepper and fatty notes. During 20–30 days of fermentation, the ROAV of β-damascenone increased to 100, replacing linalool (which decreased to 2.8–3.1), and the aroma shifted from fresh floral to rich floral. At 60 days, β-damascenone was not detected, and the overall aroma intensity was low. At 90–120 days, β-damascenone again became dominant (100), while (E,Z)-2,4-decadienal increased to 18.57, and β-ionone also increased to 12.67. In the later stage, the aroma was mainly floral, accompanied by obvious fatty and fruity notes. The aroma composition of the long slender pepper group was complex, experiencing a transition from fresh floral and green notes to rich floral and fatty notes, with enhanced aroma diversity in the later stage.

### 3.9. Microbial OTU Cluster Analysis in the Fermentation Process of Different Chili Paste Varieties

**Figure 11:**
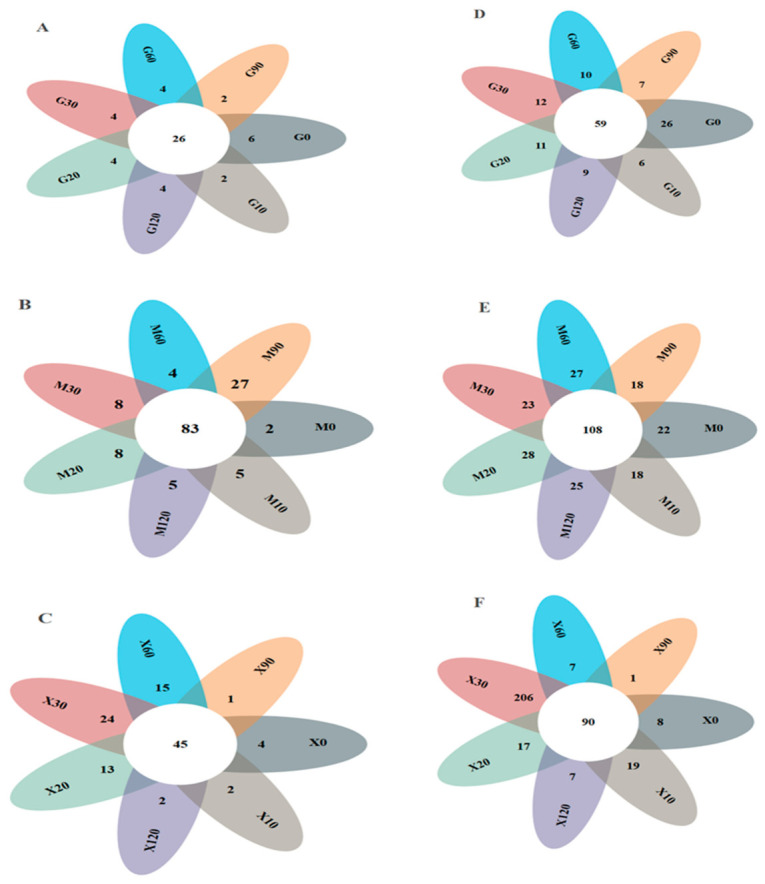
Venn diagrams showing the OTU (Operational Taxonomic Unit) distributions of bacterial (left) and fungal (right) communities during the fermentation of chopped peppers of different varieties: (**A**) Huanggong pepper; (**B**) millet pepper; (**C**) long slender pepper; (**D**) Huanggong pepper; (**E**) millet pepper; (**F**) long slender pepper.

By employing specific distance metrics to measure sequence differences or similarities, classification thresholds were determined. A distance matrix based on identical thresholds was constructed, followed by cluster analysis to ultimately form distinct Operational Taxonomic Units (OTUs) [46]. Analysis of bacterial OTU abundance during the fermentation of different varieties of chopped chili peppers yielded the results shown in Figure 11A–C.

The three chili pepper varieties shared a core set of bacterial and fungal OTUs throughout fermentation, indicating the presence of a conserved microbiome that is resilient to variety-specific differences. Bacterial OTU analysis revealed that the millet pepper group exhibited the highest number of shared OTUs across different stages (83), significantly higher than the Huanggong pepper group (26) and the long pepper group (45) (*p* < 0.05). This higher core OTU abundance suggests that millet peppers provide a more stable nutritional and physicochemical environment, which supports the persistence of a consistent bacterial community throughout fermentation.

The number of variety-specific bacterial OTUs fluctuated dynamically across groups. The Huanggong pepper group exhibited the narrowest range (2–6 OTUs), indicating a more constrained microbial recruitment process, likely due to its rapid acidification (pH drop from 5.62 to 4.26), which selectively favors acid-tolerant taxa while suppressing others. In contrast, the millet pepper group (2–27 OTUs) and long pepper group (1–24 OTUs) showed greater variability, reflecting more active community turnover. This is attributed to their more gradual pH decline and balanced nutrient profiles (higher residual sugars and amino nitrogen), which create less selective pressure and allow transient taxa to emerge at specific stages.

Fungal OTU analysis (Figure 11D–F) revealed shared OTU counts of 59, 108, and 90 for Huanggong, millet, and long peppers, respectively. The significantly higher shared OTU count in millet peppers (108) indicates a more stable and diverse fungal core community. This is likely due to the higher initial fungal load on millet pepper surfaces and its balanced carbon-to-nitrogen ratio, which supports the coexistence of multiple fungal species without competitive exclusion. The millet pepper group exhibited minimal fluctuations in unique fungal OTUs (18–28), indicating a stable fungal community structure. This stability is attributed to the consistent availability of substrates (e.g., sugars and amino acids) throughout fermentation, which prevents drastic shifts in fungal composition. The long pepper group experienced a sharp increase in unique OTUs to 206 on day 30, indicating a significant fungal community turnover. This peak coincides with the depletion of readily fermentable sugars (reducing sugar dropped from 18.45 to 6.21 g/kg by day 30), which likely triggers a shift from sugar-fermenting yeasts to more stress-tolerant filamentous fungi capable of utilizing complex carbohydrates. Throughout the fermentation process, fungal OTU counts significantly exceeded bacterial OTU counts across all three varieties, demonstrating fungal dominance in this fermentation system. This dominance is explained by the higher tolerance of fungi to the acidic environment (final pH 4.07–4.26) and their ability to produce a broader range of extracellular enzymes (e.g., pectinases and proteases), which allows them to access nutrients that bacteria cannot efficiently utilize. Consequently, fungal community dynamics exert a decisive influence on both fermentation kinetics and final product quality.

### 3.10. Analysis of Microbial Alpha Diversity During the Fermentation Process of Different Chili Pepper Varieties

**Figure 12:**
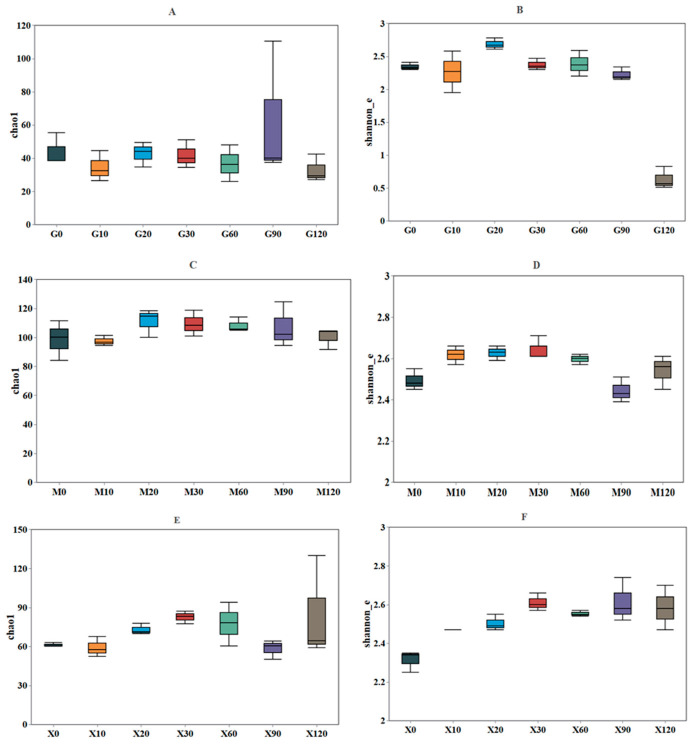
Alpha diversity indices (Chao1 and Shannon) of bacterial communities in fermented chili peppers: (**A**,**B**) Huanggong pepper; (**C**,**D**) millet pepper; (**E**,**F**) long slender pepper.

Microbial alpha diversity during the processing of different chili pepper varieties was analyzed using the Chao1 index (species richness) and Shannon index (community diversity) [47]. Results showed (Figure 12) significant differences in Chao1 indices among the three varieties (*p* < 0.05), with Mi Jiao exhibiting the highest value, followed by Xian Jiao, and Huanggong Jiao showing the lowest. This indicates that Mi Jiao peppers possessed the highest bacterial species richness, potentially related to its initial microbial composition, nutritional content, and physicochemical properties. Throughout the fermentation process, both the Chao1 and Shannon indices exhibited dynamic changes across varieties. Huanggong peppers and long peppers reached their peak values at day 20, while rice peppers peaked at day 30, indicating continuous evolution in microbial community richness and diversity in the process.

**Figure 13:**
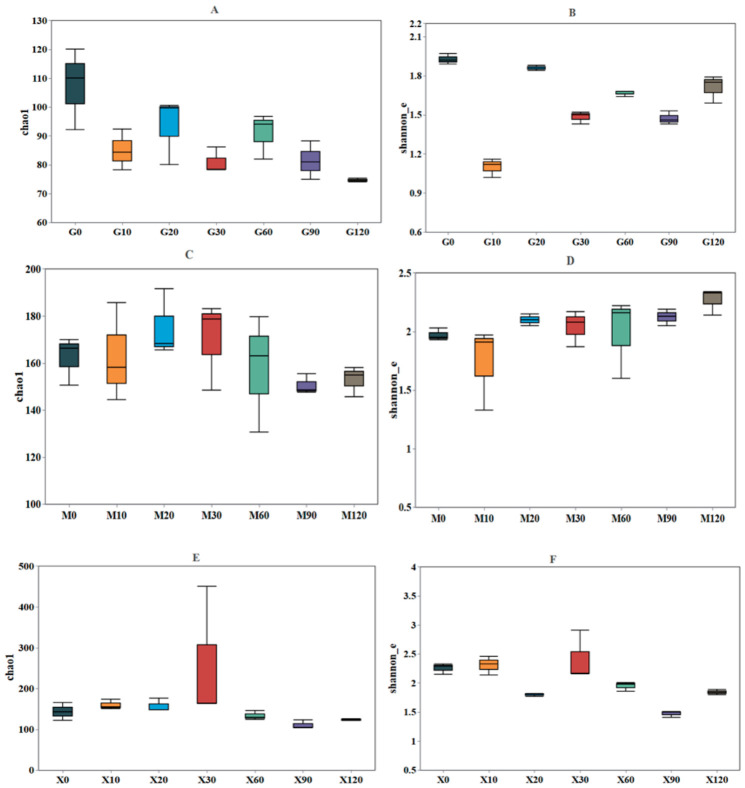
Fungal community alpha diversity indices (Chao1 and Shannon) during pepper fermentation: (**A**,**B**) Huanggong pepper; (**C**,**D**) millet pepper; (**E**,**F**) long slender pepper.

As shown in Figure 13, throughout the fermentation process, the Chao1 index of the fungal community consistently exceeded that of the bacterial community. This indicates that fungi exerted a greater influence than bacteria on the final quality of the three types of fermented chopped chili peppers, consistent with the OTU abundance analysis results. Compared to the other two chopped chili pepper groups, the Gongjiao group exhibited the lowest fungal Chao1 index throughout the fermentation process, indicating a lower fungal population during its fermentation. The Mi jiao group showed minimal fluctuation in its fungal Shannon index, suggesting a relatively stable fungal community structure in the process.

### 3.11. Analysis of Microbial Beta Diversity During the Processing of Different Chili Pepper Varieties

**Figure 14:**
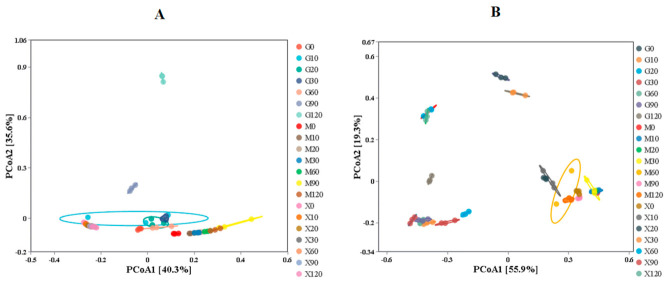
PCoA of microbial communities in fermented chili peppers: (**A**) bacterial; (**B**) fungal.

Principal Coordinate Analysis (PCoA) based on the Bray–Curtis distance was employed to assess microbial community beta diversity during the processing of different chili pepper varieties [48]. As shown in Figure 14, the first two principal components explained 75.9% and 75.2% of the variation in bacterial and fungal community structures, respectively.

The bacterial community PCoA results (Figure 14A) revealed that samples from the Mi Jiao group clustered closely at all stages, indicating similar bacterial compositions throughout fermentation. Samples from the Xian Jiao group nearly overlapped completely, suggesting relatively stable bacterial community structures. In contrast, the Huanggong Jiao group showed distinct separation between the initial and final fermentation stages, reflecting significant changes in bacterial composition during the latter phase. Furthermore, samples from the rice pepper group were closer to those of the yellow Gong pepper group than to the green pepper group, suggesting greater similarity in bacterial community structure between the former two groups.

PCoA of fungal communities (Figure 14B) revealed that samples from the rice pepper group were concentrated at each stage, indicating a relatively stable fungal community. The yellow gong pepper group and the long pepper group showed distinct separation at the start and end of fermentation, indicating significant changes in their fungal composition in the process. At the beginning of fermentation, the fungal community structures of the three groups were relatively similar.

### 3.12. Successional Changes in Microbial Community Structure During the Processing of Different Chili Pepper Varieties

**Figure 15:**
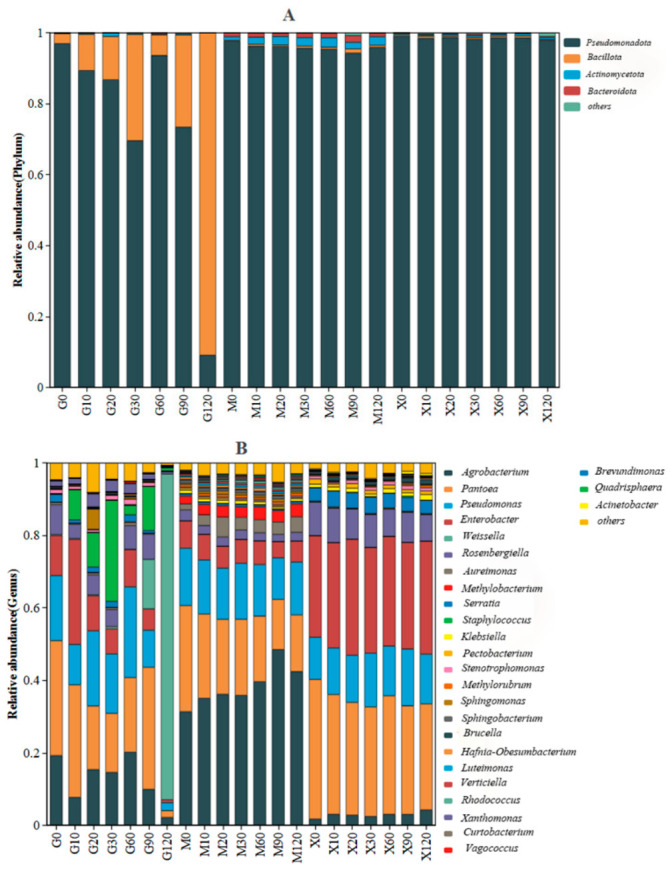
Succession of bacterial communities in different pepper varieties in the process the phylum (**A**) and genus levels (**B**).

The quality of fermented chili peppers is regulated by bacterial community structure [49]. As shown in Figure 15A, four dominant bacterial phyla—*Pseudomonadota*, *Bacillota*, *Actinomycetota*, and *Bacteroidota*—were detected across all three chili pepper varieties in the process. The dominant microbial community in the Huanggong Jiao group underwent significant succession: *Pseudomonadota* dominated at the initial fermentation stage (96.97%), while *Bacillota* became the absolute dominant group by day 120 (90.94%), with *Pseudomonadota* decreasing to 9.04%. This fundamental shift in community structure is consistent with previous findings in fermented chili products [50]. In contrast, the bacterial communities of millet and long slender peppers remained relatively stable, with *Pseudomonadota* maintaining relative abundance above 94% throughout fermentation with no significant succession of dominant phyla. The prevalence of *Pseudomonadota* in vegetable fermentations, including chili peppers [51], kimchi [52], and fermented cowpeas [53], suggests common selective pressures in vegetable-based fermentation ecosystems.

At the genus level, analysis of bacterial communities (Figure 15B) identified 174 bacterial genera across the three sample groups. Major genera with average relative abundances exceeding 1% included *Agrobacterium*, *Pantoea*, *Pseudomonas*, *Enterobacter*, and *Rosenbergiella*. *Agrobacterium* exhibited the highest abundance in the rice pepper group, while *Pantoea* and *Enterobacter* dominated in the long pepper group. Overall, the bacterial genus composition of rice and long peppers showed minimal fluctuation in the process, indicating relatively stable communities; in contrast, yellow gong peppers exhibited pronounced dynamic changes.

Analysis of unique bacterial genera revealed that the primary unique genera in rice peppers were *Aureimonas* and *Methylobacterium*, while *Klebsiella* and *Pectobacterium* dominated in long peppers. For Huanggong peppers, *Sphingomonas* and *Xanthomonas* were the dominant unique genera from days 10 to 60 of fermentation; by day 90, *Weissella* and *Glucosea* became predominant. By the end of fermentation, *Weissella* dominated with a relative abundance of 89.94%.

**Figure 16:**
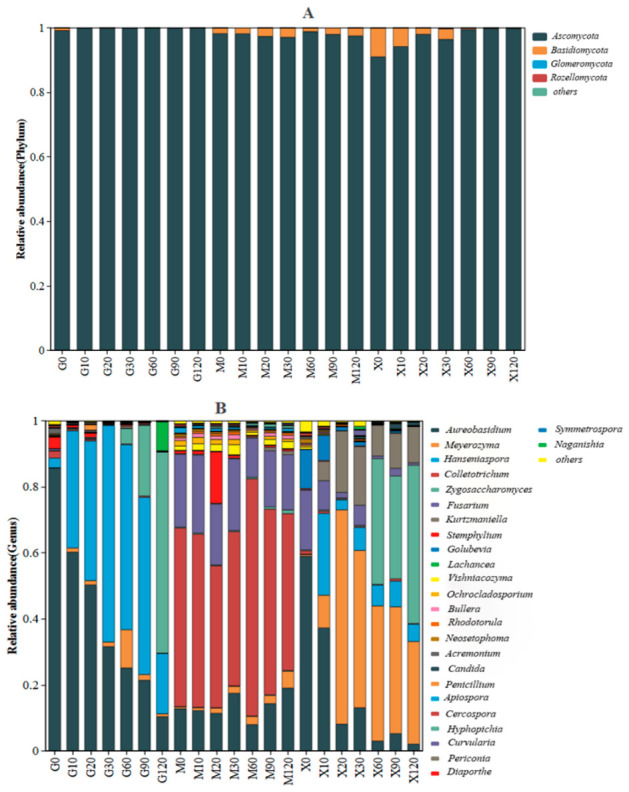
Fungal community succession during the fermentation process of multiple chopped chili pepper varieties at the phylum (**A**) and genus levels (**B**).

The fungal community structure at the phylum level is presented in Figure 16A. *Ascomycota* dominated the fungal communities across all three varieties throughout the entire fermentation process, maintaining relative abundances above 99.17% in the Huanggong pepper, 97.09% in the millet pepper, and 91.03% in the long slender pepper. This overwhelming predominance of *Ascomycota* is consistent with previous findings in fermented chili products reported by [54]. At the genus level (Figure 16B), the major shared fungal genera were *Aureobasidium* and *Meyerozyma*. *Aureobasidium* exhibited the highest average abundance (40.66%) in yellow gong peppers, gradually decreasing in the process. *Meyerozyma* showed the highest abundance (33.38%) in long peppers, exhibiting an initial increase followed by a decline.

Analysis of unique fungal genera revealed that *Hanseniaspora* and *Zygosaccharomyces* were shared between yellow gong peppers and long peppers. Among yellow gong peppers, *Hanseniaspora* exhibited a dynamic pattern of initial increase followed by a decrease; *Zygosaccharomyces* appeared in both groups starting from day 60, reaching peak abundance by fermentation completion (Huanggong peppers: 4.67–60.99%; long peppers: 31.21–48.02%). *Fusarium* was common to both rice peppers and long peppers, with abundance ranging from 11.85% to 23.67% in rice peppers and 0.71% to 18.15% in long peppers. Additionally, *Lachancea* (8.85%) appeared in Huanggong peppers at day 120. Colletotrichum was the primary characteristic genus in Mi peppers (43.05–71.94%), with *Stemphylium* (7.84%) detected at day 30. In the early fermentation stage (0–10 days), long peppers contained *Golubevia* (7.69–11.89%), while the mid-to-late stage (10–120 days) featured *Kurtzmaniella* (5.89–18.68%).

The microbial community succession during the fermentation of the three chili varieties showed significant differences. The bacterial community structure of rice peppers and long peppers remained relatively stable, with *Pseudomonadales* consistently dominating. This may be attributed to the fermentation environment (such as a gradual pH decline and balanced nutrient utilization) exerting minimal selective pressure on the initial dominant microbial communities. In contrast, Huanggong peppers underwent dramatic community succession, with the phylum *Actinomycetes* (primarily the genus *Weissella*) replacing *Pseudomonas* as the dominant group during late fermentation. This succession likely resulted from multiple factors: First, Huanggong peppers exhibited a faster acid production rate, causing rapid pH decline and creating an environment more favorable for lactic acid bacteria (like *Weissella*) while detrimental to many Gram-negative bacteria (such as *Pseudomonas*). Second, specific nutritional components in Huanggong Jiao (such as soluble sugars and amino acid composition) may favor the competitive growth of *Weissella*. Finally, microbial interactions (including competition and symbiosis) may also have facilitated this shift in dominant microbial communities. This unique pattern of microbial succession directly correlates with Huanggong Jiao’s exceptional profile of organic acids (lactic acid and acetic acid) and characteristic ester flavor compounds.

### 3.13. Correlation Analysis Between Microorganisms and Key Volatile Flavor Compounds During the Processing of Different Chili Pepper Varieties

Microbial community dynamics significantly influence the formation of key volatile flavor compounds during chili fermentation. Spearman correlation analysis was employed to investigate correlations between the top 10 most abundant microbial genera and key volatile flavor compounds.

**Figure 17:**
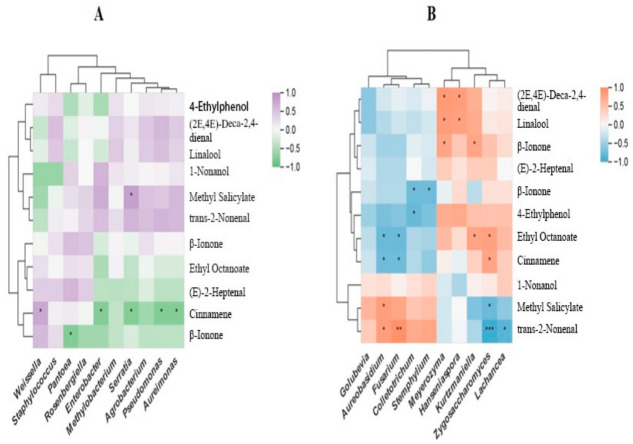
Correlation analysis between (**A**) bacterial genera, (**B**) fungal genera, and key volatile flavor compounds during the fermentation of Huanggong peppers. * indicates *p* < 0.05, ** indicates *p* < 0.01, *** indicates *p* < 0.001.

Figure 17A shows the heatmap of correlations between dominant bacterial genera and key volatile flavor compounds in the Gongjiao group. *Weissella* exhibited a significant correlation with cinnamene, which contributes woody, spicy, and peppery aromas. *Serratia* exhibited a significant positive correlation with methyl salicylate, which imparts a wintergreen oil aroma, contributing to the cool and herbal notes in chopped chili peppers.

As shown in Figure 17B, at the fungal genus level, *Aureobasidium* exhibited significant positive correlations with trans-2-nonanal and methyl salicylate. Trans-2-nonanal contributes grassy and fatty aromas. *Meyerozyma* showed significant positive correlations with (2E,4E)-dec-2,4-dienal (fatty aroma), linalool (floral), and β-ionone (violet/woody). *Hanseniaspora* showed significant positive correlations with (2E,4E)-dec-2,4-dienal and linalool. *Kurtzmaniella* exhibited significant positive correlations with β-ionone and ethyl caproate, which imparts pineapple, apple, and floral aromas. *Zygosaccharomyces* showed significant positive correlations with ethyl octanoate and caryophyllene.

**Figure 18:**
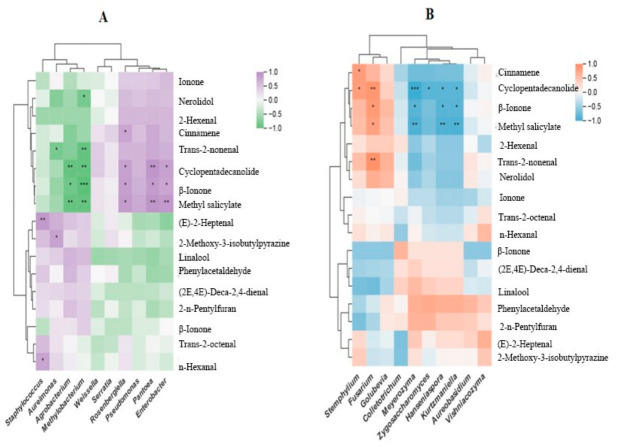
Correlation analysis between (**A**) bacterial genera, (**B**) fungal genera, and key volatile flavor compounds during the fermentation of millet peppers. * indicates *p* < 0.05, ** indicates *p* < 0.01, *** indicates *p* < 0.001.

Figure 18 shows the correlation heatmap between dominant bacterial genera and key volatile flavor compounds in the chili pepper group. As depicted in Figure 18A, at the bacterial level, *Staphylococcus* exhibited significant positive correlations with hexanal and (E)-2-heptenal, both of which contribute grassy notes. *Aureimonas* was significantly positively correlated with 2-methoxy-3-isobutylpyrazine (green pepper/earthy aroma). *Rosenbergiella* showed significant positive correlations with cinnamaldehyde, cyclopentadecanol, β-ionone, and methyl salicylate. *Pantoea* and *Enterobacter* exhibited significant positive correlations with cyclopentadecanol, β-ionone, and methyl salicylate.

As shown in Figure 18B, at the fungal genus level, *Stemphylium* exhibited significant positive correlations with cinnamaldehyde and cyclopentadecanol. *Fusarium* showed significant positive correlations with cyclopentadecanol, β-pyrocatechol, methyl salicylate, and trans-2-nonanal.

**Figure 19:**
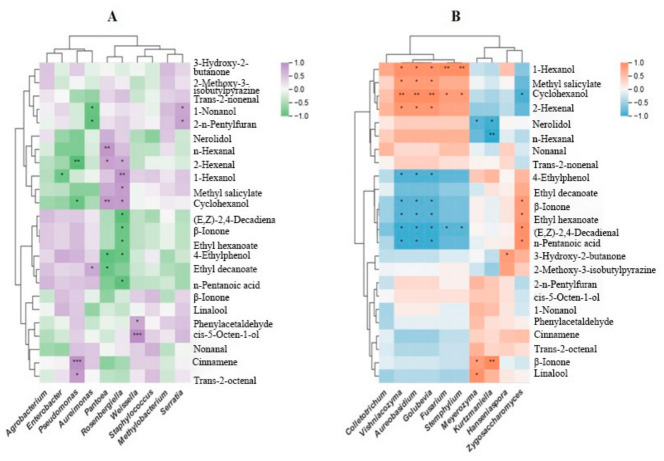
Correlation analysis between (**A**) bacterial genera, (**B**) fungal genera, and key volatile flavor compounds during the fermentation of long slender peppers. * indicates *p* < 0.05, ** indicates *p* < 0.01,*** indicates *p* < 0.001.

Figure 19 presents a heatmap correlating dominant bacterial genera with key volatile flavor compounds in the chili pepper group. As shown in Figure 19A, at the bacterial level, *Pseudomonas* exhibited significant positive correlations with cinnamaldehyde and trans-2-octenal. *Aureimonas* showed a significant positive correlation with ethyl decanoate, which contributes fruity, floral, and buttery aromas. *Pantoea* was positively correlated with hexanal, 2-hexenal, and cyclohexanol. *Rosenbergiella* showed significant positive correlations with cyclohexanol, methyl salicylate, n-hexanol, and 2-hexenal. *Weissella* exhibited significant positive correlations with cis-5-octen-1-ol and phenylacetaldehyde. The genus *Serratia* showed significant positive correlations with 2-pentylfuran and n-nonanol.

As shown in Figure 19B, at the fungal genus level, *Vishniacozyma*, *Aureobasidium*, and *Golubevia* exhibited significant correlations with 2-hexenal, cyclohexanol, methyl salicylate, and n-hexanol. *Fusarium* and *Stemphylium* showed significant positive correlations with cyclohexanol and n-hexanol. *Meyerozyma* exhibited significant positive correlations with linalool and β-ionone. *Kurtzmaniella* showed a significant positive correlation with β-ionone. *Hanseniaspora* showed a significant positive correlation with 3-hydroxy-2-butanone. *Zygosaccharomyces* exhibited significant positive correlations with n-valeric acid, (E,Z)-2,4-decadienal, ethyl n-hexanoate, and β-ionone.

## 4. Conclusions

This study systematically compared the quality and microbial dynamics of Huanggong Jiao, Mi Jiao, and Xian Jiao peppers in the process under fixed salinity conditions. It revealed the decisive influence of pepper varieties on the fermentation characteristics and flavor development of chopped chili peppers and preliminarily elucidated the underlying microbiological mechanisms. Key conclusions are as follows:

Different pepper varieties exhibit distinct fermentation patterns and quality profiles. Line peppers exhibit rapid acidification, with the fastest pH decline and reducing sugar depletion. Rice peppers demonstrate high substrate stability, retaining the highest levels of amino nitrogen and reducing sugars while exhibiting optimal color and texture stability. Huanggong peppers display strong acid-producing flavor characteristics, accumulating the highest total acidity and being the only variety to accumulate both lactic acid and acetic acid, establishing the foundation for their distinctive sour-aromatic flavor.

The formation of variety-specific flavor profiles is closely linked to key microbial community succession. Volatile flavor compound analysis reveals the following: the Huanggong Jiao variety is characterized by aldehydes, esters (e.g., ethyl caproate), and cinnamaldehyde (cinnamaldehyde); Mi Jiao is marked by exceptionally high levels of floral/woody compounds (β-ionone) and cyclopentadecanol; and long peppers exhibit a dominance of alcohol compounds, resulting in a fresher flavor profile. These characteristic flavor developments are directly linked to variety-specific microbial succession: during the late fermentation stage of Huanggong peppers, the relative abundance of *Weissella* reached 89.94%, significantly promoting the formation of cinnamonic and ester compounds; Mi Jiao exhibits diverse bacterial communities and stable fungal communities, with fungi like Bacillus anthracis positively correlated with characteristic floral compounds, whereas fungi like Saccharomyces mayeri in Xian Jiao correlate with abundant alcohol compounds.

This study systematically elucidates the formation pathways of flavor differences in chopped chili peppers from the perspective of raw material variety–microbial succession–metabolic products–sensory quality. The findings confirm that raw chili varieties profoundly influence microbial community assembly in the process through their initial physicochemical composition and microbial reservoir. This, in turn, drives differentiated metabolic activities that ultimately shape the diverse quality and flavor profiles of chopped chili peppers. These results provide a theoretical basis for targeted production of high-quality chopped chili peppers based on raw material characteristics.

Practically, for rich acid and ester aromas, the Huanggong chili is optimal when fermented into the mid-to-late stage (e.g., 60 days). For vibrant color, crisp texture, and stable flavor, the Mi chili is suitable. To achieve fresh fruit aromas and rapid flavor development, the Xian chili should undergo shorter fermentation cycles.

This study has two main limitations. First, only one salt concentration (10% *w*/*w*) was tested; future studies should examine a salinity gradient (6–12% *w*/*w*) to determine variety-specific optimal conditions. Second, the proposed role of *Weissella* in ester formation, suggested by correlation analysis, requires causal validation via inoculation experiments using pure cultures or synthetic microbial communities.

## Figures and Tables

**Table 1:** Sensory assessment standards.

Scoring Criteria and Weightings	Rating Criteria	Points/Score
Color (20%)	Bright red or golden yellow color without dullness	16~20
	Relatively red or yellow color without dullness	11~15
	Relatively red or yellow color with slight dullness	6~10
	Dark red or dark yellow color	0~5
Form (20%)	Uniform thickness with minimal juice	16~20
	Relatively uniform thickness with minimal juice	11~15
	Varied thickness with moderate juice	6~10
	Mixed thickness with abundant juice	0~5
Aroma (20%)	Characteristic fermented chili aroma	16~20
	Strong fermented chili aroma	11~15
	Mild fermented chili aroma, pronounced raw chili scent	6~10
	Pronounced spoilage odor	0~5
Crispness (20%)	Crisp and tender, good chewiness, no skin–flesh separation	16~20
	Fairly crisp and tender, good chewiness, no skin–flesh separation	11~15
	Fairly crisp and tender, average chewiness, slight skin–flesh separation	6~10
	Soft and mushy, poor chewiness, noticeable separation of skin and flesh	0~5
Flavor (20%)	Mildly sour and spicy flavor, fresh and savory	16~20
	Moderately mild sour and spicy flavor, fresh and savory	11~15
	Sour and spicy flavor not mild, pronounced raw chili pungency	6~10
	Sour and spicy flavor not mild, off-flavors present	0~5

**Table 2:** Changes in color during the fermentation process of chopped chili peppers of different varieties. where L* represents lightness and darkness, a* represents redness, and b* represents yellowness and blueness.

Fermentation Time (d)	Group	L*	a*	b*
0	G10	51.03 ± 0.56 ^a^	10.52 ± 0.52 ^c^	30.76 ± 1.29 ^a^
	M10	45.66 ± 0.27 ^b^	29.72 ± 0.79 ^a^	19.76 ± 0.72 ^b^
	X10	44.63 ± 0.34 ^c^	27.42 ± 0.24 ^b^	16.65 ± 0.53 ^c^
10	G10	50.46 ± 0.18 ^a^	9.88 ± 0.32 ^c^	29.84 ± 0.41 ^a^
	M10	44.64 ± 1.07 ^b^	28.28 ± 1.37 ^a^	18.99 ± 0.37 ^b^
	X10	41.06 ± 0.42 ^c^	26.12 ± 0.4 ^b^	15.17 ± 0.28 ^c^
20	G10	49.96 ± 0.58 ^a^	9.52 ± 0.41 ^c^	28.91 ± 0.3 ^a^
	M10	44.09 ± 0.51 ^b^	26.28 ± 0.38 ^a^	17.76 ± 0.37 ^b^
	X10	40.51 ± 0.14 ^c^	24.44 ± 0.74 ^b^	14.15 ± 0.3 ^c^
30	G10	48.85 ± 0.61 ^a^	9.3 ± 0.31 ^c^	28.31 ± 0.51 ^a^
	M10	43.29 ± 0.24 ^b^	25.72 ± 0.55 ^a^	16.22 ± 0.67 ^b^
	X10	39.79 ± 0.64 ^c^	22.99 ± 0.29 ^b^	13.11 ± 0.36 ^c^
60	G10	47.46 ± 0.47 ^a^	9.25 ± 0.58 ^c^	25.49 ± 1.16 ^a^
	M10	42.07 ± 0.11 ^b^	24.87 ± 0.6 ^a^	18.14 ± 0.25 ^b^
	X10	39.28 ± 0.29 ^c^	21.27 ± 0.4 ^b^	14.25 ± 0.64 ^c^
90	G10	46.04 ± 0.35 ^a^	9.03 ± 0.36 ^c^	22.81 ± 0.74 ^a^
	M10	44.59 ± 1.23 ^a^	22.86 ± 0.88 ^a^	17.47 ± 0.67 ^b^
	X10	38.79 ± 0.68 ^b^	20.46 ± 0.32 ^b^	13.7 ± 0.19 ^c^
120	G10	44.99 ± 0.38 ^a^	8.67 ± 0.4 ^c^	20.98 ± 0.58 ^a^
	M10	42.4 ± 0.37 ^b^	21.74 ± 0.26 ^a^	15.81 ± 0.23 ^b^
	X10	39.78 ± 0.67 ^c^	17.69 ± 0.63 ^b^	13.2 ± 1.25 ^c^

Note: In the same column, different letters (e.g., a, b, c) indicate significant differences between different groups at the same fermentation time (*p* < 0.05), while the same letter indicates no significant difference.

**Table 3:** Volatile flavor compounds with OAV > 1 during Huanggong pepper fermentation.

Name	Threshold (μg/kg)	OAV						
		0 d	10 d	20 d	30 d	60 d	90 d	120 d
Methyl salicylate	40.00	10.32 ± 0.45 ^a^	7.20 ± 0.31 ^b^	4.11 ± 0.22 ^c^	8.39 ± 0.38 ^ab^	4.22 ± 0.24 ^c^	2.83 ± 0.15 ^cd^	1.47 ± 0.08 ^d^
Ethyl octanoate	19.30	/	/	/	/	1.64 ± 0.09 ^b^	3.70 ± 0.18 ^a^	1.11 ± 0.06 ^b^
Caryophyllene	3.60	/	/	/	/	/	18.26 ± 0.82 ^a^	2.76 ± 0.14 ^b^
β-Ionone	0.07	/	/	/	363.23 ± 15.21 ^a^	/	/	18.39 ± 0.93 ^b^
β-Phellandrene	0.01	/	/	/	/	1736.10 ± 72.43 ^b^	20,949.41 ± 862.37 ^a^	/
(2E,4E)-Dec-2,4-dienal	0.03	679.46 ± 28.32 ^f^	2261.60 ± 93.45 ^e^	3402.03 ± 0.22 ^c^	3352.82 ± 139.64 ^c^	4414.20 ± 183.76 ^a^	2832.08 ± 117.84 ^d^	1974.36 ± 82.27 ^e^
trans-2-Nonanal	0.19	357.11 ± 14.86 ^b^	239.90 ± 9.97 ^c^	801.70 ± 33.40 ^a^	332.38 ± 13.85 ^b^	140.98 ± 5.87 ^d^	93.21 ± 3.88 ^e^	15.66 ± 0.65 ^f^
(E)-2-Heptenal	4.60	/	/	/	/	/	5.44 ± 0.27	/
4-Ethylphenol	13.00	/	/	<1	1.84 ± 0.09 ^bc^	2.39 ± 0.12 ^a^	1.06 ± 0.05 ^c^	1.72 ± 0.08 ^b^
Linalool	2.40	14.05 ± 0.62 ^e^	19.47 ± 0.86 ^d^	62.79 ± 2.77 ^a^	51.53 ± 2.28 ^b^	74.04 ± 3.26 ^a^	45.87 ± 2.02 ^bc^	30.71 ± 1.35 ^cd^
n-Nonanol	4.55	1.70 ± 0.08 ^b^	5.72 ± 0.28 ^a^	<1	<1	3.03 ± 0.15 ^ab^	<1	1.25 ± 0.06 ^b^

Note: “/” indicates not detected; “<1” means OAV less than 1. Data are expressed as the mean ± standard deviation (SD, n = 3). Different lowercase letters in the same row denote significant differences at *p* < 0.05 via one-way ANOVA followed by Duncan’s multiple range test.

**Table 4:** Volatile flavor compounds with OAV > 1 during millet pepper fermentation.

Name	Threshold (μg/kg)	OAV						
		0 d	10 d	20 d	30 d	60 d	90 d	120 d
β-Ionone	0.01	11,791.79 ± 473.67 ^a^	7909.75 ± 316.39 ^bc^	7764.92 ± 310.60 ^bc^	8765.45 ± 350.62 ^b^	6369.58 ± 254.78 ^cd^	6974.84 ± 278.99 ^c^	3670.51 ± 146.82 ^d^
(2E,4E)-Dec-2,4-dienal	0.03	/	1420.73 ± 56.83 ^d^	2761.95 ± 110.48 ^c^	3112.89 ± 124.52 ^bc^	3703.08 ± 148.12 ^a^	2941.56 ± 117.66 ^bc^	1324.94 ± 52.99 ^d^
trans-2-Nonanal	0.19	155.86 ± 6.23 ^a^	86.53 ± 3.46 ^b^	37.59 ± 1.50 ^c^	75.90 ± 3.04 ^b^	/	64.59 ± 2.58 ^b^	22.73 ± 0.91 ^c^
trans-2-Octenal	0.34	107.14 ± 4.29 ^bc^	64.98 ± 2.60 ^c^	231.48 ± 9.26 ^a^	317.69 ± 12.71 ^a^	134.03 ± 5.36 ^b^	192.00 ± 7.68 ^ab^	/
Linalool	2.40	14.67 ± 0.59 ^d^	13.79 ± 0.55 ^d^	19.40 ± 0.78 ^c^	27.85 ± 1.11 ^bc^	45.02 ± 1.80 ^a^	31.84 ± 1.27 ^b^	18.00 ± 0.72 ^cd^
Methyl salicylate	40.00	12.14 ± 0.49 ^a^	9.35 ± 0.37 ^b^	8.98 ± 0.36 ^bc^	9.57 ± 0.38 ^b^	5.12 ± 0.20 ^d^	4.69 ± 0.19 ^d^	2.29 ± 0.09 ^e^
Nerol	10.00	10.77 ± 0.43 ^a^	6.35 ± 0.25 ^c^	6.18 ± 0.25 ^c^	7.04 ± 0.28 ^bc^	4.92 ± 0.20 ^d^	6.44 ± 0.26 ^c^	3.09 ± 0.12 ^e^
Caryophyllene	3.60	8.41 ± 0.34 ^a^	4.19 ± 0.17 ^bc^	7.19 ± 0.29 ^ab^	3.28 ± 0.13 ^c^	2.96 ± 0.12 ^c^	1.56 ± 0.06 ^d^	3.29 ± 0.13 ^c^
Cyclopentadecanolide	1.80	6.18 ± 0.25 ^a^	2.87 ± 0.11 ^b^	2.68 ± 0.11 ^b^	1.79 ± 0.07 ^c^	/	/	/
Phenylacetaldehyde	6.30	<1	2.22 ± 0.09 ^bc^	1.78 ± 0.07 ^c^	2.87 ± 0.11 ^ab^	2.11 ± 0.08 ^bc^	3.60 ± 0.14 ^a^	2.23 ± 0.09 ^bc^
(E)-2-Heptenal	4.60	/	1.29 ± 0.05 ^b^	3.55 ± 0.14 ^a^	1.34 ± 0.05 ^b^	/	1.46 ± 0.06 ^b^	1.59 ± 0.06 ^b^
Ionone	10.60	1.64 ± 0.07 ^bc^	1.09 ± 0.04 ^c^	1.54 ± 0.06 ^bc^	2.08 ± 0.08 ^b^	3.96 ± 0.16 a	/	<1
2-Hexenal	30.00	1.75 ± 0.07	/	/	/	/	/	/
2-Pentylfuran	5.80	/	/	/	2.25 ± 0.09 ^a^	<1	1.68 ± 0.07 ^b^	<1
n-Hexanal	5.00	/	/	2.21 ± 0.09 ^b^	6.20 ± 0.25 ^a^	/	<1	/
2-Methoxy-3-isobutylpyrazine	0.38	/	/	39.28 ± 1.57 ^a^	/	/	/	22.60 ± 0.90 ^b^
β-Ionone	0.07	/	/	/	/	2097.82 ± 83.91	/	/

Note: “/” indicates not detected; “<1” means OAV less than 1. Data are expressed as the mean ± standard deviation (SD, n = 3). Different lowercase letters in the same row denote significant differences at *p* < 0.05 via one-way ANOVA followed by Duncan’s multiple range test. Single-time-point-detected compounds are only marked with SD without letters.

**Table 5:** Volatile flavor compounds with OAV > 1 during long slender pepper fermentation.

Name	Threshold (μg/kg)	OAV						
		0 d	10 d	20 d	30 d	60 d	90 d	120 d
β-Ionone	0.01	/	/	5238.80 ± 209.55 ^a^	3070.63 ± 122.83 ^b^	/	2407.40 ± 96.30 ^bc^	980.31 ± 39.21 ^c^
(E,Z)-2,4-Decadienal	0.04	/	/	/	/	404.32 ± 16.17 ^b^	134.54 ± 5.38 ^c^	182.08 ± 7.28 ^a^
β-Ionone	0.07	/	/	/	/	218.63 ± 8.75 ^a^	143.08 ± 5.72 ^b^	124.23 ± 4.97 ^b^
Linalool	2.40	24.51 ± 0.98 ^d^	94.62 ± 3.78 ^b^	146.58 ± 5.86 ^a^	95.10 ± 3.80 ^b^	130.36 ± 5.21 ^a^	102.90 ± 4.12 ^b^	47.16 ± 1.89 ^c^
Ethyl hexanoate	2.20	/	/	/	/	22.66 ± 0.91 ^a^	20.99 ± 0.84 ^a^	7.54 ± 0.30 ^b^
Ethyl decanoate	5.00	/	/	/	/	/	<1	1.63 ± 0.07
n-Pentanoic acid	280.00	/	/	/	/	/	<1	1.47 ± 0.06
Caryophyllene	3.60	/	/	/	28.44 ± 1.14 ^a^	2.37 ± 0.09 ^b^	29.18 ± 1.17 ^a^	1.38 ± 0.06 ^b^
4-Ethylphenol	13.00	/	/	1.10 ± 0.04 ^a^	<1	<1	1.74 ± 0.07 ^a^	1.33 ± 0.05 ^a^
Methyl salicylate	40.00	3.75 ± 0.15 ^bc^	5.09 ± 0.20 ^a^	4.73 ± 0.19 ^ab^	3.65 ± 0.15 ^bc^	2.10 ± 0.08 ^d^	1.75 ± 0.07 ^d^	1.24 ± 0.05 ^e^
n-Nonanol	4.55	/	1.74 ± 0.07 ^a^	1.85 ± 0.07 ^a^	1.04 ± 0.04 ^b^	<1	/	<1
Nerol	10.00	<1	1.60 ± 0.06	/	/	<1	/	<1
3-Hydroxy-2-butanone	14.00	/	<1	/	<1	11.89 ± 0.48	<1	<1
trans-2-Octenal	0.34	/	/	34.91 ± 1.40 ^c^	67.68 ± 2.71 ^b^	73.32 ± 2.93 ^ab^	108.46 ± 4.34 ^a^	/
2-Methoxy-3-isobutylpyrazine	0.38	/	72.31 ± 2.89 ^a^	/	/	42.19 ± 1.69 ^b^	36.99 ± 1.48 ^b^	/
Nonanal	1.10	11.89 ± 0.48 ^b^	8.28 ± 0.33 ^bc^	/	14.93 ± 0.60 ^a^	8.49 ± 0.34 ^bc^	35.97 ± 1.44 ^a^	/
n-Hexanol	5.60	29.42 ± 1.18 ^ab^	33.04 ± 1.32 ^a^	22.20 ± 0.89 ^bc^	31.50 ± 1.26 ^a^	19.93 ± 0.80 ^c^	26.89 ± 1.08 ^b^	/
n-Hexanal	5.00	5.68 ± 0.23 ^a^	4.07 ± 0.16 ^b^	/	/	2.29 ± 0.09 ^c^	/	/
2-n-Pentylfuran	5.80	/	2.20 ± 0.09 ^a^	2.32 ± 0.09 ^a^	1.50 ± 0.06 ^b^	1.26 ± 0.05 ^b^	/	/
Phenylethylaldehyde	6.30	/	/	/	1.53 ± 0.06 ^a^	1.11 ± 0.04 ^b^	/	/
Cyclohexanol	90.00	2.12 ± 0.08 ^a^	<1	<1	<1	<1	/	/
cis-5-Octen-1-ol	6.00	/	/	/	2.43 ± 0.10	/	/	/
trans-2-Nonanal	0.19	/	65.78 ± 2.63 ^a^	55.90 ± 2.24 ^b^	/	/	/	/
2-Hexenal	30.00	2.08 ± 0.08 ^a^	<1	<1	/	/	/	/

Note: “/” indicates not detected; “<1” means OAV less than 1. Data are expressed as the mean ± standard deviation (SD, n = 3). Different lowercase letters in the same row denote significant differences at *p* < 0.05 via one-way ANOVA followed by Duncan’s multiple range test. Single-time-point-detected compounds are only marked with SD without letters.

**Table 6:** ROVA values (%) of the Huanggong pepper group during the fermentation process.

Name	0 d	10 d	20 d	30 d	60 d	90 d	120 d
Methyl salicylate	1.52 ± 0.07 ^a^	0.32 ± 0.02 ^b^	0.12 ± 0.01 ^c^	0.25 ± 0.01 ^bc^	0.10 ± 0.01 ^c^	0.01 ± 0.00 ^d^	0.01 ± 0.00 ^d^
Ethyl octanoate	/	/	/	/	0.04 ± 0.00 ^b^	0.02 ± 0.00 ^b^	0.01 ± 0.00 ^a^
Caryophyllene	/	/	/	/	/	0.09 ± 0.00 ^a^	0.01 ± 0.00 ^b^
β-Ionone	/	/	/	10.83 ± 0.48 ^a^	/	/	0.09 ± 0.00 ^b^
β-Phellandrene	/	/	/	/	39.33 ± 1.74 ^b^	100.00 ± 0.00 ^a^	/
(2E,4E)-Dec-2,4-dienal	100.00 ± 0.00 ^a^	100.00 ± 0.00 ^a^	100.00 ± 0.00 ^a^	100.00 ± 0.00 ^a^	100.00 ± 0.00 ^a^	13.52 ± 0.60 ^b^	9.43 ± 0.42 ^c^
trans-2-Nonanal	52.56 ± 2.32 ^a^	10.61 ± 0.47 ^b^	23.56 ± 1.04 ^b^	9.91 ± 0.44 ^bc^	3.19 ± 0.14 ^cd^	0.44 ± 0.02 ^de^	0.07 ± 0.00 ^e^
(E)-2-Heptenal	/	/	/	/	/	0.03 ± 0.00	/
4-Ethylphenol	/	/	<0.01	0.05 ± 0.00 ^a^	0.05 ± 0.00 ^a^	0.01 ± 0.00 ^b^	0.01 ± 0.00 ^b^
Linalool	2.07 ± 0.09 ^a^	0.86 ± 0.04 ^b^	1.85 ± 0.08 ^a^	1.54 ± 0.07 ^a^	1.68 ± 0.07 ^a^	0.22 ± 0.01 ^c^	0.15 ± 0.01 ^c^
n-Nonanol	0.25 ± 0.01 ^a^	0.25 ± 0.01 ^a^	<0.01	<0.01	0.07 ± 0.00 ^b^	<0.01	0.01 ± 0.00 ^b^

Note: The maximum value in each column is set to 100%. “/” indicates not detected. Data are expressed as the mean ± standard deviation (SD, n = 3). Different lowercase letters in the same row indicate significant differences at *p* < 0.05.

**Table 7:** ROVA values (%) of the millet pepper group during the fermentation process.

Name	0 d	10 d	20 d	30 d	60 d	90 d	120 d
β-Ionone	100.00 ± 0.00 ^a^	100.00 ± 0.00 ^a^	100.00 ± 0.00 ^a^	100.00 ± 0.00 ^a^	100.00 ± 0.00 ^a^	100.00 ± 0.00 ^a^	100.00 ± 0.00 ^a^
(2E,4E)-Dec-2,4-dienal	/	17.96 ± 0.72 ^d^	35.57 ± 1.42 ^c^	35.51 ± 1.42 ^c^	58.14 ± 2.33 ^a^	42.18 ± 1.69 ^b^	36.10 ± 1.44 ^c^
trans-2-Nonanal	1.32 ± 0.05 ^a^	1.09 ± 0.04 ^ab^	0.48 ± 0.02 ^c^	0.87 ± 0.03 ^b^	/	0.93 ± 0.04 ^b^	0.62 ± 0.02 ^c^
trans-2-Octenal	0.91 ± 0.04 ^bc^	0.82 ± 0.03 ^c^	2.98 ± 0.12 ^a^	3.62 ± 0.14 ^a^	2.10 ± 0.08 ^b^	2.75 ± 0.11 ^ab^	/
Linalool	0.12 ± 0.00 ^d^	0.17 ± 0.01 ^cd^	0.25 ± 0.01 ^bc^	0.32 ± 0.01 ^b^	0.71 ± 0.03 ^a^	0.46 ± 0.02 ^b^	0.49 ± 0.02 ^b^
Methyl salicylate	0.10 ± 0.00 ^a^	0.12 ± 0.00 ^a^	0.12 ± 0.00 ^a^	0.11 ± 0.00 ^a^	0.08 ± 0.00 ^b^	0.07 ± 0.00 ^b^	0.06 ± 0.00 ^b^
Nerol	0.09 ± 0.00 ^a^	0.08 ± 0.00 ^ab^	0.08 ± 0.00 ^ab^	0.08 ± 0.00 ^ab^	0.08 ± 0.00 ^ab^	0.09 ± 0.00 ^a^	0.08 ± 0.00 ^ab^
Caryophyllene	0.07 ± 0.00 ^a^	0.05 ± 0.00 ^ab^	0.09 ± 0.00 ^a^	0.04 ± 0.00 ^b^	0.05 ± 0.00 ^ab^	0.02 ± 0.00 ^c^	0.09 ± 0.00 ^a^
Cyclopentadecanolide	0.05 ± 0.00 ^ab^	0.04 ± 0.00 ^b^	0.03 ± 0.00 ^bc^	0.02 ± 0.00 ^c^	/	/	/
Phenylacetaldehyde	<0.01	0.030.00 ^bc^	0.02 ± 0.00 ^c^	0.030.00 ^bc^	0.03 ± 0.00 ^bc^	0.05 ± 0.00 ^ab^	0.06 ± 0.00 ^a^
(E)-2-Heptenal	/	0.02 ± 0.00 ^c^	0.05 ± 0.00 ^ab^	0.02 ± 0.00 ^c^	/	0.02 ± 0.00 ^c^	0.04 ± 0.00 ^a^
Ionone	0.01 ± 0.00 ^b^	0.01 ± 0.00 ^b^	0.02 ± 0.00 ^c^	0.02 ± 0.00 ^c^	0.06 ± 0.00 ^a^	/	<0.01
2-Hexenal	0.01 ± 0.00 ^b^	/	/	/	/	/	/
2-Pentylfuran	/	/	/	0.03 ± 0.00 ^bc^	<0.01	0.02 ± 0.00 ^c^	<0.01
n-Hexanal	/	/	0.03 ± 0.00 ^bc^	0.07 ± 0.00 ^a^	/	<0.01	/
2-Methoxy-3-isobutylpyrazine	/	/	0.51 ± 0.02 ^b^	/	/	/	0.62 ± 0.02 ^a^
β-Ionone	/	/	/	/	32.92 ± 1.32	/	/

Note: The maximum value in each column is set to 100%. “/” indicates not detected. Data are expressed as the mean ± standard deviation (SD, n = 3). Different lowercase letters in the same row indicate significant differences at *p* < 0.05. Single-time-point-detected compounds are only marked with SD without letters.

**Table 8:** ROVA values (%) of the long slender pepper group during the fermentation process.

Name	0 d	10 d	20 d	30 d	60 d	90 d	120 d
β-Ionone	/	/	100.00 ± 0.00 ^a^	100.00 ± 0.00 ^a^	/	100.00 ± 0.00 ^a^	100.00 ± 0.00 ^a^
(E,Z)-2,4-Decadienal	/	/	/	/	3.10 ± 0.12 ^b^	5.59 ± 0.22 ^b^	18.57 ± 0.74 ^a^
β-Ionone	/	/	/	/	1.68 ± 0.07 ^a^	5.94 ± 0.24 ^b^	12.67 ± 0.51 ^c^
Linalool	100.00 ± 0.00 ^a^	100.00 ± 0.00 ^a^	2.80 ± 0.11 ^c^	3.10 ± 0.12 ^b^	1.00 ± 0.04 ^d^	4.27 ± 0.17 ^bc^	4.81 ± 0.19 ^b^
Ethyl hexanoate	/	/	/	/	0.17 ± 0.01 ^a^	0.87 ± 0.03 ^a^	0.77 ± 0.03 ^b^
Ethyl decanoate	/	/	/	/	/	<0.01	0.17 ± 0.01
n-Pentanoic acid	/	/	/	/	/	<0.01	0.15 ± 0.01
Caryophyllene	/	/	/	0.93 ± 0.04 ^a^	0.02 ± 0.00 ^b^	1.21 ± 0.05 ^a^	0.14 ± 0.01 ^b^
4-Ethylphenol	/	/	0.02 ± 0.00 ^a^	<0.01	<0.01	0.07 ± 0.00 ^a^	0.14 ± 0.01 ^a^
Methyl salicylate	15.30 ± 0.61 ^a^	5.38 ± 0.22 ^b^	0.09 ± 0.00 ^c^	0.12 ± 0.00 ^c^	0.02 ± 0.00 ^d^	0.07 ± 0.00 ^cd^	0.13 ± 0.01 ^c^
n-Nonanol	/	1.84 ± 0.07 ^a^	0.04 ± 0.00 ^a^	0.03 ± 0.00 ^b^	<0.01	/	<0.01
Nerol	<0.01	1.69 ± 0.07	/	/	<0.01	/	<0.01
3-Hydroxy-2-butanone	/	<0.01	/	<0.01	0.09 ± 0.00	<0.01	<0.01
trans-2-Octenal	/	/	0.67 ± 0.03 ^c^	2.20 ± 0.09 ^b^	0.56 ± 0.02 ^bc^	4.50 ± 0.18 a	/
2-Methoxy-3-isobutylpyrazine	/	76.43 ± 3.06 a	/	/	0.32 ± 0.01 ^b^	1.54 ± 0.06 ^b^	/
Nonanal	48.53 ± 1.94 ^b^	8.75 ± 0.35 ^bc^	/	0.49 ± 0.02 ^a^	0.07 ± 0.00 ^bc^	1.49 ± 0.06 ^a^	/
n-Hexanol	120.07 ± 4.80 ^ab^	34.92 ± 1.40 ^a^	0.42 ± 0.02 ^bc^	1.03 ± 0.04 ^a^	0.15 ± 0.01 ^c^	1.12 ± 0.04 ^b^	/
n-Hexanal	23.18 ± 0.93 ^a^	4.30 ± 0.17 ^b^	/	/	0.02 ± 0.00 ^c^	/	/
2-n-Pentylfuran	/	2.32 ± 0.09 ^a^	0.04 ± 0.09 ^a^	0.05 ± 0.00 ^b^	0.01 ± 0.00 ^b^	/	/
Phenylethylaldehyde	/	/	/	0.05 ± 0.00 ^a^	0.01 ± 0.00 ^b^	/	/
Cyclohexanol	8.65 ± 0.35 ^a^	<0.01	<0.01	<0.01	<0.01	/	/
cis-5-Octen-1-ol	/	/	/	0.08 ± 0.00	/	/	/
trans-2-Nonanal	/	69.53 ± 2.78 ^a^	1.07 ± 0.04 ^b^	/	/	/	/
2-Hexenal	8.49 ± 0.34 ^a^	<0.01	<0.01	/	/	/	/

Note: The maximum value in each column is set to 100%. “/” indicates not detected. Data are expressed as the mean ± standard deviation (SD, n = 3). Different lowercase letters in the same row indicate significant differences at *p* < 0.05. Single-time-point-detected compounds are only marked with SD without letters.

## Data Availability

The original contributions presented in the study are included in the article; further inquiries can be directed to the corresponding author.

## Supplementary Materials

The following supporting information can be downloaded at: https://www.mdpi.com/article/10.3390/foods15111970/s1, Table S1 Changes in Organic Acid Content During Fermentation in G10 Group; Table S2 Changes in Organic Acid Content During Fermentation in M10 Group; Table S3 Changes in Organic Acid Content During Fermentation in X10 Group; Table S4 Changes in Organic Acid Content During Fermentation in G15 Group; Table S5 Changes in Organic Acid Content During Fermentation in G20 Group; Table S6 Changes in Free Amino Acid Content During Fermentation in G10 Group; Table S7 Changes in Free Amino Acid Content During Fermentation in M10 Group; Table S8 Changes in Free Amino Acid Content During Fermentation in X10 Group; Table S9 Changes in Free Amino Acid Content During Fermentation in G15 Group.
