# Supplementary material for: Effects of Different Chili Pepper Varieties on the Quality and Microbial Diversity of Spontaneously Fermented Chili Paste
Source: Foods. 2026 Jun 2;15(11):1970. doi: 10.3390/foods15111970 (PMC13257216; doi:10.3390/foods15111970)
Supplement: Supplementary file 1 [file foods-15-01970-s001.zip › foods-4325323-supplementary.pdf]

Table S1 Changes in Organic Acid Content During Fermentation in G10 Group

Types of Organic Acids (mg/g)	Fermentation time/d						
	0	10	20	30	60	90	120
Oxalic acid	0.058±0.007^{b}	0.022±0.002^{e}	0.024±0.003^{e}	ND^{f}	0.047±0.002^{c}	0.038±0.003^{d}	0.087±0.003^{a}
Tartaric acid	0.75±0.016^{e}	1.506±0.029^{b}	1.725±0.021^{a}	1.28±0.018^{c}	0.551±0.032^{f}	1.109±0.011^{d}	1.258±0.068^{c}
Malic acid	0.125±0.027^{e}	0.074±0.052^{f}	0.169±0.005^{e}	1.034±0.005^{c}	0.386±0.041^{d}	1.316±0.029^{a}	1.134±0.007^{b}
Lactic acid	ND^{c}	ND^{c}	ND^{c}	ND^{c}	ND^{c}	0.791±0.048^{b}	1.835±0.009^{a}
Acetic acid	ND^{c}	ND^{c}	ND^{c}	ND^{c}	ND^{c}	0.111±0.017^{a}	0.076±0.045^{b}
Citric acid	1.184±0.077^{b}	1.291±0.01^{a}	0.852±0.002^{c}	0.753±0.04^{d}	0.219±0.013^{g}	0.31±0.018^{f}	0.566±0.028^{e}
Succinic acid	0.214±0.031^{c}	0.261±0.014^{b}	0.334±0.008^{a}	0.231±0.013^{c}	0.331±0.022^{a}	ND^{d}	ND^{d}
Total content	2.332±0.103^{e}	3.153±0.05^{d}	3.105±0.014^{d}	3.298±0.045^{c}	1.534±0.025^{f}	3.676±0.043^{b}	4.954±0.08^{a}

Table S2 Changes in Organic Acid Content During Fermentation in M10 Group

Types of Organic Acids (mg/g)	Fermentation time/d						
	0	10	20	30	60	90	120
Oxalic acid	0.185±0.006b	0.214±0.019a	0.184±0.002b	0.206±0.012a	0.118±0.004c	0.069±0.003d	0.055±0.007d
Tartaric acid	0.725±0.002e	1.609±0.054c	1.933±0.021a	1.618±0.003c	1.822±0.011b	1.445±0.007d	1.588±0.044c
Malic acid	0.153±0.009g	0.569±0.015e	0.324±0.021f	1.016±0.035d	1.443±0.012c	1.735±0.012a	1.543±0.064b
Lactic acid	ND	ND	ND	ND	ND	ND	ND
Acetic acid	NDf	0.533±0.018a	0.345±0.013c	0.41±0.006b	0.152±0.003e	0.232±0.011d	0.222±0.008d
Citric acid	0.217±0.007e	0.915±0.001c	0.911±0.015c	1.053±0.002b	1.075±0.006a	0.757±0.028d	0.927±0.012c
Succinic acid	0.637±0.051a	0.147±0.103e	0.209±0.014de	0.613±0.005ab	0.248±0.012d	0.422±0.008c	0.562±0.011b
Total	1.917±0.047f	3.987±0.13d	3.906±0.021e	4.916±0.057a	4.859±0.022ab	4.66±0.037c	4.897±0.112a

Table S3 Changes in Organic Acid Content During Fermentation in X10 Group

Types of Organic Acids (mg/g)	Fermentation time/d						
	0	10	20	30	60	90	120
Oxalic acid	0.033±0.003^{a}	0.018±0.005^{b}	0.006±0.002^{c}	ND^{d}	ND^{d}	ND^{d}	ND^{d}
Tartaric acid	0.798±0.013^{f}	1.475±0.052^{d}	1.526±0.035^{c}	1.81±0.031^{b}	1.86±0.038^{a}	1.347±0.005^{e}	1.48±0.009^{cd}
Malic acid	0.108±0.004^{g}	0.447±0.016^{e}	0.682±0.011^{c}	0.65±0.021^{d}	0.279±0.013^{f}	1.353±0.006^{a}	0.81±0.018^{b}
Lactic acid	ND	ND	ND	ND	ND	ND	ND
Acetic acid	ND^{d}	ND^{d}	0.482±0.006^{b}	0.519±0.037^{a}	ND^{d}	0.108±0.022^{c}	ND^{d}
Citric acid	0.765±0.095^{c}	0.116±0.017^{f}	0.636±0.035^{de}	1.244±0.006^{a}	1.034±0.116^{b}	0.586±0.011^{e}	0.693±0.012^{cd}
Succinic acid	0.31±0.007^{e}	1.862±0.031^{a}	1.868±0.043^{a}	0.363±0.006^{d}	0.642±0.011^{b}	0.239±0.004^{f}	0.475±0.009^{c}

Types of Organic Acids (mg/g)	Fermentation time/d						
	0	10	20	30	60	90	120
Total content	2.013±0.085^{g}	3.917±0.145^{e}	5.021±0.088^{a}	4.587±0.073^{b}	3.816±0.097^{c}	3.633±0.016^{d}	3.459±0.028^{f}

Table S4 Changes in Organic Acid Content During Fermentation in G15 Group

Types of Organic Acids (mg/g)	Fermentation time/d						
	0	10	20	30	60	90	120
Oxalic acid	0.05±0.001^{d}	0.036±0.003^{e}	0.029±0.001^{f}	0.018±0.005^{g}	0.089±0.003^{c}	0.117±0.004^{b}	0.144±0.003^{a}
Tartaric acid	0.72±0.016^{d}	1.03±0.104^{c}	1.307±0.061^{a}	1.101±0.009^{bc}	0.164±0.005^{e}	0.672±0.031^{d}	1.123±0.015^{b}
Malic acid	0.12±0.003^{e}	ND^{f}	ND^{f}	0.833±0.092^{b}	0.328±0.004^{d}	0.586±0.069^{c}	0.949±0.014^{a}
Lactic acid	ND^{c}	ND^{c}	ND^{c}	ND^{c}	ND^{c}	0.511±0.025^{b}	0.808±0.016^{a}
Acetic acid	ND^{b}	ND^{b}	ND^{b}	ND^{b}	ND^{b}	0.406±0.527^{a}	ND^{b}
Citric acid	1.06±0.019^{a}	0.313±0.006^{cd}	0.423±0.015^{b}	0.424±0.006^{b}	0.339±0.004^{c}	0.282±0.003^{d}	0.308±0.016^{cd}
Succinic acid	0.19±0.005^{c}	0.191±0.002^{c}	0.185±0.003^{c}	0.192±0.049^{c}	ND^{d}	0.244±0.054^{b}	1.062±0.003^{a}
Total content	2.145±0.026^{c}	1.57±0.108^{e}	1.943±0.044^{d}	2.568±0.123^{bc}	0.919±0.006^{f}	2.818±0.555^{b}	4.39±0.036^{a}

Table S5 Changes in Organic Acid Content During Fermentation in G20 Group

Types of Organic Acids (mg/g)	Fermentation time/d						
	0	10	20	30	60	90	120
Oxalic acid	0.048±0.004^{a}	0.049±0.001^{a}	0.032±0.002^{bc}	0.035±0.002^{b}	0.029±0.001^{d}	0.024±0.007^{e}	0.016±0.002^{f}
Tartaric acid	0.68±0.012^{c}	0.809±0.008^{b}	0.438±0.382^{d}	0.635±0.006^{cd}	1.332±0.014^{a}	0.788±0.005^{b}	0.614±0.002^{cd}
Malic acid	0.16±0.004^{f}	0.109±0.004^{g}	0.259±0.002^{e}	0.334±0.014^{d}	0.381±0.011^{c}	0.424±0.007^{b}	0.705±0.009^{a}
Lactic acid	ND	ND	ND	ND	ND	ND	ND
Acetic acid	ND^{b}	ND^{b}	ND^{b}	ND^{b}	ND^{b}	0.108±0.019^{a}	ND^{b}
Citric acid	0.92±0.019^{a}	0.388±0.021^{d}	0.472±0.006^{c}	0.405±0.008^{d}	0.631±0.002^{b}	0.214±0.009^{e}	0.141±0.009^{f}
Succinic acid	0.18±0.007^{c}	0.223±0.013^{b}	ND^{d}	ND^{d}	0.283±0.016^{a}	ND^{d}	ND^{d}

Succinic acid	1.979±0.024^{b}	1.577±0.038^{c}	1.201±0.383^{d}	1.41±0.024^{cd}	2.655±0.017^{a}	1.558±0.026^{c}	1.48±0.015^{c}

Table S6 Changes in Free Amino Acid Content During Fermentation in G10 Group

Types of Free Amino Acids (mg/g)	Fermentation time/d						
	0	10	20	30	60	90	120
Valine	0.174±0.021^{b}	0.126±0.006^{c}	0.163±0.008^{b}	0.108±0.007^{c}	0.156±0.014^{b}	0.203±0.021^{a}	0.199±0.002^{a}
Methionine	ND^{e}	0.051±0.008^{d}	0.145±0.009^{b}	0.117±0.004^{c}	0.149±0.003^{b}	0.172±0.005^{a}	0.172±0.004^{a}
Isoleucine	ND^{d}	ND^{d}	0.117±0.006^{a}	0.088±0.005^{c}	0.101±0.006^{bc}	0.109±0.002^{ab}	0.109±0.024^{ab}
Tyrosine	0.327±0.018^{e}	0.238±0.008^{f}	0.675±0.021^{a}	0.565±0.02^{b}	0.522±0.018^{c}	0.425±0.022^{d}	0.42±0.003^{d}
Phenylalanine	0.083±0.004^{e}	0.118±0.012^{d}	0.191±0.012^{b}	0.154±0.006^{c}	0.192±0.007^{b}	0.222±0.018^{a}	0.219±0.004^{a}
Histidine	ND^{e}	0.388±0.035^{c}	0.765±0.023^{a}	0.639±0.038^{b}	0.806±0.039^{a}	0.271±0.015^{d}	ND^{e}
Arginine	0.183±0.008^{e}	0.201±0.012^{d}	0.338±0.007^{b}	0.265±0.007^{c}	0.34±0.004^{b}	0.358±0.016^{a}	0.349±0.004^{ab}
Bitter amino acids	0.767±0.029^{g}	1.121±0.037^{f}	2.394±0.028^{a}	1.935±0.068^{c}	2.266±0.052^{b}	1.76±0.047^{d}	1.468±0.026^{e}
Threonine	0.951±0.014^{d}	1.016±0.054^{c}	1.19±0.011^{ab}	0.908±0.013^{d}	1.153±0.052^{b}	1.215±0.059^{a}	1.204±0.019^{ab}
Serine	1.682±0.038^{e}	2.532±0.021^{c}	2.789±0.01^{a}	2.151±0.022^{d}	2.671±0.047^{ab}	2.551±0.2^{bc}	2.49±0.017^{c}
Glycine	0.527±0.009^{c}	0.723±0.04^{b}	0.829±0.013^{a}	0.706±0.06^{b}	0.799±0.014^{a}	0.321±0.024^{d}	0.266±0.02^{e}
Alanine	0.138±0.007^{c}	0.14±0.003^{c}	0.148±0.011^{c}	0.122±0.007^{d}	0.16±0.007^{b}	0.18±0.005^{a}	0.181±0.008^{a}
Sweet amino acids	3.298±0.055^{f}	4.412±0.099^{c}	4.956±0.008^{a}	3.887±0.046^{e}	4.784±0.016^{b}	4.266±0.211^{cd}	4.142±0.055^{d}
Aspartic acid	0.147±0.008^{e}	0.25±0.02^{d}	0.289±0.01^{c}	0.233±0.006^{d}	0.331±0.013^{a}	0.315±0.006^{ab}	0.312±0.009^{b}
Glutamic acid	ND^{d}	ND^{d}	0.187±0.003^{c}	ND^{d}	0.211±0.007^{b}	0.245±0.01^{a}	0.252±0.012^{a}
Umami amino acids	0.147±0.008^{e}	0.25±0.02^{d}	0.476±0.008^{c}	0.233±0.006^{d}	0.542±0.019^{b}	0.561±0.008^{ab}	0.564±0.01^{a}
Cysteine	0.225±0.047^{e}	0.308±0.008^{d}	0.594±0.018^{a}	0.421±0.032^{c}	0.498±0.018^{b}	0.522±0.005^{b}	0.525±0.007^{b}
Lysine	0.086±0.003^{d}	0.095±0.003^{d}	0.183±0.014^{ab}	0.139±0.003^{c}	0.169±0.006^{b}	0.187±0.021^{a}	0.192±0.002^{a}
Proline	0.29±0.036^{c}	0.3±0.022^{bc}	0.43±0.004^{a}	0.339±0.003^{b}	0.438±0.002^{a}	0.445±0.057^{a}	0.463±0.006^{a}
Tasteless Amino Acids	0.602±0.048^{f}	0.704±0.022^{e}	1.207±0.02^{a}	0.899±0.038^{d}	1.104±0.023^{c}	1.153±0.041^{bc}	1.18±0.002^{ab}
Total Content	4.814±0.007^{g}	6.487±0.153^{f}	9.033±0.053^{a}	6.954±0.081^{e}	8.696±0.103^{b}	7.74±0.285^{c}	7.356±0.072^{d}

Table S7 Changes in Free Amino Acid Content During Fermentation in M10 Group

Free Amino Acids (mg/g)	Fermentation time/d						
	0	10	20	30	60	90	120
Valine	0.102±0.003^{f}	0.149±0.002^{e}	0.166±0.002^{d}	0.182±0.004^{c}	0.216±0.012^{b}	0.228±0.019^{ab}	0.232±0.013^{a}
Methionine	0.058±0.004^{e}	0.102±0.01^{d}	0.107±0.011^{cd}	0.116±0.006^{bc}	0.122±0.005^{b}	0.145±0.008^{a}	0.106±0.009^{cd}
Isoleucine	ND	ND	ND	ND	ND	ND	ND
Tyrosine	0.433±0.011^{a}	0.324±0.003^{b}	0.308±0.013^{b}	0.205±0.016^{c}	0.176±0.01^{d}	0.191±0.025^{cd}	0.145±0.018^{e}
Phenylalanine	0.121±0.012^{cd}	0.132±0.003^{bc}	0.135±0.004^{b}	0.134±0.003^{b}	0.153±0.007^{a}	0.144±0.003^{ab}	0.116±0.012^{d}
Histidine	0.607±0.013^{ab}	0.621±0.011^{a}	0.595±0.012^{b}	0.53±0.006^{c}	0.497±0.017^{d}	ND^{e}	ND^{e}
Arginine	0.595±0.011^{a}	0.265±0.007^{b}	0.223±0.01^{d}	0.222±0.013^{d}	0.249±0.006^{c}	0.225±0.005^{d}	0.219±0.003^{d}
Bitter amino acids	1.916±0.043^{a}	1.593±0.023^{b}	1.534±0.019^{c}	1.389±0.035^{d}	1.411±0.047^{d}	0.933±0.054^{e}	0.818±0.021^{f}

Free Amino Acids (mg/g)	Fermentation time/d						
	0	10	20	30	60	90	120
Threonine	0.411±0.007^{e}	0.814±0.006^{d}	0.847±0.011^{c}	0.852±0.006^{c}	0.933±0.012^{b}	0.987±0.052^{a}	0.954±0.007^{ab}
Serine	2.125±0.02^{a}	1.61±0.046^{c}	1.66±0.029^{b}	1.617±0.009^{bc}	1.616±0.019^{bc}	1.536±0.036^{d}	1.65±0.037^{bc}
Glycine	0.764±0.005^{a}	0.605±0.013^{b}	0.611±0.017^{b}	0.585±0.017^{c}	0.536±0.007^{d}	0.254±0.01^{e}	0.223±0.011^{f}
Alanine	0.251±0.005^{a}	0.169±0.003^{c}	0.176±0.007^{c}	0.178±0.004^{c}	0.195±0.004^{b}	0.192±0.013^{b}	0.178±0.003^{c}
Sweet amino acids	3.551±0.033^{a}	3.198±0.06^{c}	3.294±0.014^{b}	3.232±0.026^{bc}	3.28±0.041^{b}	2.969±0.052^{d}	3.005±0.035^{d}
Aspartic acid	0.166±0.009^{bc}	0.135±0.009^{e}	0.157±0.008^{cd}	0.152±0.003^{d}	0.211±0.009^{a}	0.174±0.008^{b}	0.166±0.008^{bc}
Glutamic acid	ND	ND	ND	ND	ND	ND	ND
Umami amino acids	0.166±0.009^{bc}	0.135±0.009^{e}	0.157±0.008^{cd}	0.152±0.003^{d}	0.211±0.009^{a}	0.174±0.008^{b}	0.166±0.008^{bc}
Cysteine	0.337±0.009^{ab}	0.366±0.011^{ab}	0.388±0.058^{a}	0.339±0.008^{ab}	0.365±0.006^{ab}	0.347±0.061^{ab}	0.306±0.015^{b}
Lysine	0.086±0.004^{d}	0.106±0.002^{c}	0.114±0.006^{bc}	0.118±0.011^{abc}	0.127±0.005^{a}	0.124±0.012^{ab}	0.116±0.005^{abc}
Proline	0.254±0.002^{c}	0.28±0.004^{b}	0.292±0.002^{ab}	0.294±0.002^{ab}	0.31±0.006^{a}	0.284±0.02^{b}	0.28±0.017^{b}
Odorless amino acids	0.678±0.009^{c}	0.753±0.015^{ab}	0.794±0.066^{a}	0.751±0.021^{ab}	0.802±0.013^{a}	0.755±0.044^{ab}	0.703±0.029^{bc}
Total content	6.311±0.07^{a}	5.677±0.08^{b}	5.779±0.098^{b}	5.524±0.05^{c}	5.705±0.107^{b}	4.831±0.113^{d}	4.692±0.038^{e}

Table S8 Changes in Free Amino Acid Content During Fermentation in X10 Group

Types of Free Amino Acids (mg/g)	Fermentation time/d						
	0	10	20	30	60	90	120
Valine	0.072±0.003^{e}	0.115±0.012^{c}	0.095±0.004^{d}	0.134±0.017^{b}	0.146±0.003^{b}	0.164±0.006^{a}	0.134±0.007^{b}
Methionine	ND^{e}	0.087±0.008^{d}	0.088±0.004^{d}	0.104±0.005^{bc}	0.108±0.002^{b}	0.123±0.006^{a}	0.098±0.004^{c}
Isoleucine	ND^{d}	0.063±0.003^{b}	0.065±0.003^{b}	0.081±0.001^{a}	0.069±0.002^{b}	0.068±0.006^{b}	0.044±0.006^{c}
Tyrosine	0.61±0.017^{a}	0.509±0.017^{b}	0.51±0.015^{b}	0.482±0.008^{c}	0.248±0.003^{e}	0.283±0.003^{d}	0.205±0.009^{f}
Phenylalanine	0.081±0.004^{d}	0.117±0.003^{c}	0.115±0.005^{c}	0.136±0.002^{b}	0.137±0.004^{b}	0.159±0.007^{a}	0.121±0.005^{c}
Histidine	0.208±0.01^{b}	0.471±0.014^{a}	0.175±0.002^{c}	0.207±0.01^{b}	ND^{d}	ND^{d}	ND^{d}
Arginine	0.177±0.005^{d}	0.236±0.003^{b}	0.231±0.003^{b}	0.25±0.002^{a}	0.256±0.008^{a}	0.26±0.018^{a}	0.199±0.002^{c}
Bitter amino acids	1.147±0.013^{d}	1.598±0.015^{a}	1.279±0.024^{c}	1.393±0.008^{b}	0.965±0.016^{f}	1.057±0.039^{e}	0.735±0.117^{g}
Threonine	0.718±0.005^{d}	0.787±0.006^{b}	0.75±0.003^{c}	0.832±0.031^{a}	0.853±0.016^{a}	0.598±0.018^{e}	0.762±0.015^{bc}
Serine	1.385±0.02^{cd}	1.435±0.021^{b}	1.367±0.018^{d}	1.4±0.003^{c}	1.43±0.013^{b}	1.502±0.015^{a}	1.234±0.015^{e}
Glycine	0.525±0.005^{b}	0.543±0.011^{a}	0.465±0.006^{c}	0.456±0.007^{c}	0.239±0.003^{d}	0.211±0.008^{e}	0.168±0.004^{f}
Alanine	0.072±0.004^{f}	0.105±0.012^{de}	0.102±0.004^{e}	0.127±0.007^{bc}	0.133±0.01^{b}	0.148±0.006^{a}	0.115±0.007^{cd}
Sweet amino acids	2.7±0.02^{c}	2.871±0.032^{a}	2.684±0.025^{cd}	2.815±0.041^{b}	2.655±0.013^{d}	2.458±0.025^{e}	2.279±0.006^{f}
Aspartic acid	0.151±0.003^{e}	0.201±0.005^{d}	0.219±0.013^{cd}	0.23±0.004^{bc}	0.246±0.007^{b}	0.274±0.015^{a}	0.218±0.025^{cd}
Glutamic acid	ND^{e}	0.126±0.006^{d}	0.13±0.003^{d}	0.152±0.005^{c}	0.163±0.005^{b}	0.185±0.01^{a}	0.134±0.006^{d}
Umami amino acids	0.151±0.003^{e}	0.328±0.007^{d}	0.349±0.016^{d}	0.382±0.009^{c}	0.409±0.01^{b}	0.459±0.021^{a}	0.351±0.031^{d}
Cysteine	0.218±0.021^{d}	0.356±0.004^{b}	0.351±0.006^{b}	0.421±0.002^{a}	0.366±0.011^{b}	0.403±0.021^{a}	0.316±0.003^{c}
Lysine	0.074±0.004^{d}	0.106±0.007^{c}	0.103±0.008^{c}	0.118±0.005^{b}	0.12±0.005^{b}	0.146±0.005^{a}	0.125±0.011^{b}
Proline	0.088±0.006^{e}	0.124±0.008^{d}	0.125±0.003^{d}	0.146±0.009^{bc}	0.155±0.002^{b}	0.175±0.007^{a}	0.141±0.004^{c}
Non-umami amino acids	0.379±0.022^{e}	0.585±0.007^{d}	0.58±0.018^{d}	0.685±0.007^{b}	0.641±0.014^{c}	0.724±0.032^{a}	0.582±0.013^{d}
Total content	4.377±0.052^{d}	5.383±0.023^{a}	4.892±0.054^{b}	5.274±0.05^{a}	4.671±0.033^{c}	4.699±0.115^{c}	3.946±0.103^{e}

Table S9 Changes in Free Amino Acid Content During Fermentation in G15 Group

Types of Free Amino Acids (mg/g)	Fermentation time/d						
	0	10	20	30	60	90	120
Valine	0.09±0.002^{d}	0.159±0.003^{a}	0.132±0.002^{b}	ND^{e}	0.049±0.002^{c}	0.054±0.004^{c}	0.054±0.003^{c}
Methionine	ND	ND	ND	ND	ND	ND	ND
Isoleucine	ND	ND	ND	ND	ND	ND	ND
Tyrosine	0.405±0.022^{b}	0.474±0.067^{a}	0.495±0.022^{a}	0.401±0.015^{b}	0.396±0.005^{bc}	0.387±0.005^{bc}	0.351±0.013^{cd}
Phenylalanine	0.083±0.004^{c}	0.117±0.006^{ab}	0.127±0.013^{a}	0.038±0.002^{d}	0.106±0.01^{b}	0.109±0.011^{b}	0.11±0.005^{b}
Histidine	ND^{c}	ND^{c}	0.349±0.031^{b}	ND^{c}	ND^{c}	ND^{c}	0.469±0.007^{a}
Arginine	0.177±0.005^{b}	0.265±0.007^{a}	0.189±0.008^{b}	0.116±0.005^{d}	0.141±0.008^{c}	0.149±0.008^{c}	0.151±0.002^{c}
Bitter amino acids	0.754±0.021^{d}	1.015±0.057^{c}	1.292±0.057^{a}	0.555±0.015^{f}	0.692±0.01^{e}	0.698±0.021^{e}	1.134±0.016^{b}
Threonine	0.621±0.013^{b}	0.894±0.009^{a}	0.883±0.018^{a}	0.494±0.004^{d}	0.598±0.006^{c}	0.612±0.003^{bc}	0.631±0.005^{b}
Serine	1.393±0.1^{b}	1.996±0.036^{a}	2.025±0.04^{a}	1.142±0.019^{e}	1.338±0.027^{d}	1.377±0.014^{c}	1.388±0.028^{b}
Glycine	0.332±0.061^{b}	0.666±0.012^{a}	0.676±0.013^{a}	0.331±0.024^{b}	0.35±0.023^{b}	0.335±0.003^{b}	0.323±0.003^{c}
Alanine	0.05±0.002^{e}	0.085±0.002^{b}	0.093±0.003^{a}	0.059±0.004^{d}	0.076±0.003^{c}	0.08±0.003^{b}	0.084±0.003^{b}
Sweet-tasting amino acid	2.396±0.174^{c}	3.641±0.034^{a}	3.677±0.073^{a}	2.026±0.044^{e}	2.363±0.055^{d}	2.404±0.023^{c}	2.426±0.027^{b}
Aspartic acid	0.143±0.006^{d}	0.221±0.014^{a}	0.232±0.011^{a}	0.121±0.003^{e}	0.154±0.003^{c}	0.158±0.007^{c}	0.172±0.007^{b}
Glutamic acid	ND	ND	ND	ND	ND	ND	ND
Umami amino acid	0.143±0.006^{d}	0.221±0.014^{a}	0.232±0.011^{a}	0.121±0.003^{e}	0.154±0.003^{c}	0.158±0.007^{c}	0.172±0.007^{b}
Cysteine	0.204±0.01^{d}	0.345±0.018^{a}	0.319±0.008^{a}	0.214±0.016^{d}	0.262±0.007^{b}	0.273±0.006^{b}	0.276±0.002^{b}
Lysine	0.063±0.004^{d}	0.104±0.003^{a}	0.109±0.003^{a}	0.079±0.002^{c}	0.098±0.012^{ab}	0.103±0.011^{a}	0.104±0.013^{a}
Proline	0.231±0.005^{c}	0.334±0.011^{b}	0.361±0.008^{a}	0.182±0.031^{e}	0.221±0.012^{d}	0.229±0.009^{c}	0.235±0.002^{c}
Tasteless amino acid	0.497±0.009^{c}	0.782±0.008^{a}	0.788±0.017^{a}	0.475±0.046^{c}	0.582±0.019^{b}	0.605±0.019^{b}	0.616±0.011^{b}
Total content	3.792±0.198^{d}	5.659±0.101^{b}	5.989±0.145^{a}	3.177±0.021^{e}	3.79±0.063^{d}	3.865±0.043^{d}	4.348±0.03^{c}
